# Selective Capture of Anti‐*N*‐glucosylated *NTHi* Adhesin Peptide Antibodies by a Multivalent Dextran Conjugate

**DOI:** 10.1002/cbic.202100515

**Published:** 2021-12-06

**Authors:** Antonio Mazzoleni, Feliciana Real‐Fernandez, Francesca Nuti, Roberta Lanzillo, Vincenzo Brescia Morra, Paolo Dambruoso, Monica Bertoldo, Paolo Rovero, Jean‐Maurice Mallet, Anna Maria Papini

**Affiliations:** ^1^ Laboratoire des Biomolécules Département de Chimie École Normale Supérieure PSL University Sorbonne Université CNRS 24 rue Lhomond 75005 Paris France; ^2^ Interdepartmental Research Unit of Peptide and Protein Chemistry and Biology Department of Chemistry “Ugo Schiff” University of Florence Via della Lastruccia 13 50019 Sesto Fiorentino Italy; ^3^ Multiple Sclerosis Clinical Care and Research Centre Department of Neurosciences Reproductive Sciences and Odontostomatology Federico II University Via Pancini 5 80131 Naples Italy; ^4^ ISOF – Istituto per la Sintesi Organica e la Fotoreattività Consiglio Nazionale delle Ricerche Via Gobetti 101 40129 Bologna Italy; ^5^ Dipartimento di Scienze chimiche, farmaceutiche ed agrarie Università di Ferrara Via Fossato di Mortara 17 44121 Ferrara Italy; ^6^ Interdepartmental Research Unit of Peptide and Protein Chemistry and Biology Department of Neurosciences, Psychology, Drug Research and Child Health Section of Pharmaceutical Sciences and Nutraceutics University of Florence Via Ugo Schiff 6 50019 Sesto Fiorentino Italy

**Keywords:** antibody caption, dextran conjugates, ELISA, multivalence, peptides

## Abstract

Tentacle‐like polymers decorated with several copies of peptide antigens can be interesting tools for increasing the ability to capture circulating antibodies in patient sera, using cooperative effects for stronger avidity. We previously showed that antibodies from multiple sclerosis (MS) patient sera preferentially recognize hyperglucosylated adhesin protein HMW1ct of non‐typeable *Haemophilus influenzae* (*NTHi*). We selected the C‐terminal HMW1ct(1347–1354) minimal epitope and prepared the diglucosylated analogue Ac‐KAN(Glc)VTLN(Glc)TTG‐K(N_3_)‐NH_2_ to graft a 40 kDa dextran scaffold modified with glycidyl‐propargyl moieties to perform a copper catalyzed alkyne‐azide coupling reaction (CuAAC). Quantitative NMR measurements allowed the characterization of the peptide loading (19.5 %) on the multivalent dextran conjugate. This novel polymeric structure displayed optimal capturing properties of both IgG and, more interestingly, IgM antibodies in MS sera. Specific antibodies from a representative MS serum, were successfully depleted using a Sepharose resin bearing the new glucosylated multivalent conjugate, as confirmed by ELISA. These results may offer a promising proof‐of‐concept for the selective purification of high affinity autoantibodies from sera of autoimmune patients, in general, and of specific high affinity antibodies against a minimally glcosylated epitope Asn(Glc) from sera of multiple sclerosis (MS) patients, in particular.

## Graphical abstract

**Dextran‐based tentacles** were decorated with peptide antigens as a way to increase the ability to capture circulating IgM antibodies in multiple sclerosis patient sera as disease biomarkers. A novel peptide‐dextran conjugate bearing multiple copies of a selected epitope of the non‐typeable *Haemophilus Influenzae* adhesin protein was synthesis and characterized. Immunological experiments provide the proof‐of concept for the identification and capture of specific IgM antibodies in patient sera.

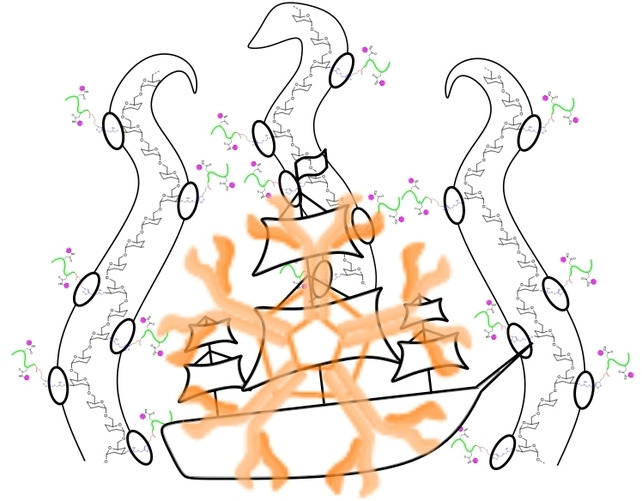

## Introduction

Peptides mimicking relevant epitopes, once conjugated to tentacle‐like polymer scaffolds, can benefit of synergistic physicochemical multivalent interactions favoring antibody recognition. Therefore, suitable polymers grafted with peptide epitopes constitute a promising class of soft materials potentially useful for a multitude of innovative and elegant applications.[^1^, ^2^] Historically, polymeric scaffolds based on carbohydrate epitopes have been used as immunosorbents, acting as “autoantibody scavengers” to remove pathogenic antibodies, particularly IgMs, from patient sera.[^3^, ^4^, ^5^] Various grafting strategies enable control of the overall architecture of the final grafted polymer, integrating precise chemical structures with diverse functionalities, yielding novel synthetic polymeric multivalent conjugates. The stability and processability of these bioinspired polymers enable their use as surrogates of the mimicked molecules, featuring similar functions and occasionally exceeding the performance because of multivalent presentation.^[1]^ The era of “click”‐type chemistries,[^6^, ^7^] i. e., copper catalyzed alkyne‐azide coupling reaction (CuAAC), oxime, Staudinger ligation, thiol‐ene, Glaser reaction, and many more bioorthogonal and biomimetic coupling reactions, promoted the access to complex and well‐defined peptide‐polymer conjugates.

Exploitation of peptides and in particular glycopeptides reproducing antigens recognizing antibodies, holds the great potential to answer many unsolved questions in chemical immunology.^[8]^ In fact, thanks to the ability of peptides to mimic specific portions of protein antigens, several examples of “peptide mimics” have been used as synthetic immunological probes.^[9]^ In this perspective, the development of synthetic probes interacting with high affinity specific autoantibodies, biomarkers of autoimmune diseases, can contribute to set up diagnostic/prognostic peptide‐based immunoassays particularly useful to guide therapeutic treatments.^[10]^ Moreover, microarchitectures displaying peptide antigens as multivalent antibody ligands at high density can act as molecular “baits” to fish out specific autoantibodies from patient sera. Therefore, multivalent peptide conjugates can be efficient stationary phases for specific immunoaffinity columns to deplete specific autoimmune antibodies from sera.

Branched polymers with a predefined structure are usually based on repetitive addition of glycopeptides to a central core molecule. Therefore, this backbone can be exploited as scaffold to produce tentacle‐like three‐dimensional structures (3D) presenting linear peptide antigens in a multivalent fashion. These conjugated structures reproduce “natural” peptide antigen presentation, thus increasing antibody recognition for IgM antibodies. In fact, while IgG‐type antibodies can be conveniently purified by affinity chromatography, IgM purification still presents unsolved problems, mostly due to IgMs pentameric spatial orientation. To the best of our knowledge, no specific peptide ligands able to capture IgMs from autoimmune patient sera have been described, particularly when both IgG‐ and IgM‐isotype antibodies are present. This is possibly because of the highest affinity of IgGs for antigens. This issue could be overcome exploiting IgMs higher avidity thanks to their pentameric structure.

Consequently, the development of a tentacle‐like structure presenting peptide antigens able to recognize circulating antibodies in sera may constitute a promising strategy to face the unmet need of diagnostic and prognostic tools, and for autoantibody removal in most of the immune‐mediated diseases. Additionally, the quantification of IgMs can improve the stratification of patients in an early stage of the disease, hypothesizing to be reminiscent of bacterial infection triggering an immune response before the onset of the disease.

Novel materials, exposing multiple copies of peptide antigens in a suitable 3D orientation, will be propaedeutic to develop selective apheresis techniques to deplete pathogenic antibodies in patients affected by autoimmune diseases.^[11]^ In this context, we previously developed a synthetic multiple antigenic peptide, carrying the minimal glucosylated epitope Asn(Glc) anchored to a 19‐atoms PEG‐based spacer, as probe to reveal specific and high affinity anti‐Asn(Glc) antibodies in Multiple Sclerosis (MS).^[12]^ Since the simple Asn(Glc) moiety is not represented in the human glycoproteome repertoire, we turned our attention to bacteria, in particular to non‐typeable *Haemophilus influenzae* (*NTHi*), which was reported to express cell‐surface adhesins including N‐Glc, to establish a connection between *NTHi* infection and MS.^[13]^ In fact, we found that antibodies to anti‐hyperglucosylated *NTHi* adhesin protein HMW1ct(N‐Glc) (bearing multiple glucosylated epitopes on asparagine residues) were present in a subpopulation of MS patients. This was the first insight that a native multivalent Asn‐glucosylated bacterial antigen was involved in MS.^[13]^

As a result of this ground‐breaking finding, we aimed to develop a novel peptide‐based dextran conjugate derived from *NTHi* HMW1ct(N‐Glc) to detect and possibly to capture antibodies in patient sera. To fulfill this goal, the dendrimer scaffold should meet some essential requirements. Dextran is a high molecular weight, inert, water‐soluble polymer that has been widely used in a variety of biomedical applications.[^14^, ^15^, ^16^, ^17^] It is mainly constituted of linear α(1‐6)linked glucopyranosides with minor amount of α(1‐3) linkages accounting for dextran branching. Therefore, the remaining free hydroxyl functions can be easily functionalized to bind peptide epitopes by either direct attachment or through a linker.^[17]^ The synthetic strategy for the construction of dextran‐based tentacles requires three steps: 1) functionalization of the polymer with moieties able to yield a selective bioorthogonal reaction; 2) functionalization of the relevant peptide epitopes with a complementary reactive partner motif; 3) coupling reaction between the two components.

We report herein a general and efficient method to produce a tentacle‐like dextran‐based polymer functionalized with *NTHi* glucopeptides, showing high affinity toward anti‐Asn(Glc) antibodies, taking advantage of avidity effect. In particular, we describe the synthesis and characterization of a peptide antigen‐based dendrimer containing capturing‐tentacles and its antibody (IgM and IgG) binding capacity and capturing properties.

## Results and Discussion

### Epitope selection

We previously demonstrated that N‐Glc containing epitopes in non‐typeable *Haemophilus influenzae* adhesin C‐terminal portion HMW1ct(1205–1526) are essential for high‐affinity antibody binding in a subpopulation of MS patients.^[13]^

With the aim of developing shorter peptide probes maintaining binding to these antibodies, we developed and tested differently glucosylated peptide analogs of the minimal fragment HMW1ct(1347–1354). In particular, we showed that the di‐N‐glucosylated adhesin peptide Ac‐KAN(Glc)VTLN(Glc)TT‐NH_2_ is able to inhibit high‐avidity binding of IgM antibodies to the parent glucosylated bacterial adhesin.^[18]^

This peptide contains two spatially close N‐glucosylation sites and an additional lysine residue at the N‐terminus (Table 1, Peptide 1). We also synthesized three glucosylated analogs bearing: i) N‐Glc moiety linked to Asn1348 (Table 1, Peptide 1’_A_) ii) N‐Glc moiety linked to Asn1352 (Table 1, Peptide 1’_B_), and iii) two N‐Glc moieties, on both glucosylation sites (Table 1, Peptide 1’_A,B_). Moreover, we modified Peptide 1’_A,B_ elongating the sequence at C‐terminus with GK(N_3_) to obtain the Peptide 1′_A,B_GK(ϵN_3_) (Ac‐KAN(Glc)VTLN(Glc)TTGK(N_3_)‐NH_2_), containing an azide moiety to allow grafting to an alkyne functionalized dextran backbone by a selective bioorthogonal copper catalyzed alkyne‐azide coupling reaction (CuAAC). We extended the sequence of Peptide 1′_A,B_ with G1355 to obtain a short spacer between the glucosylated sites and the grafting moiety. All peptides were synthesized in solid‐phase by a microwave‐assisted Fmoc/tBu strategy.

**Table 1:** Peptide sequences.

Sequence	Short name	
ANVTLNTT	HMW1ct(1347–1354)	
Ac‐KANVTLNTT‐NH_2_	[Ac‐Lys1346]HMW1ct(1346–1354)‐NH_2_	Peptide 1
Ac‐KAN(Glc)VTLNTT‐NH_2_	[Ac‐Lys1346;Asn(Glc)1348]HMW1ct(1346–1354)‐NH_2_	Peptide 1’_A_
Ac‐KANVTLN(Glc)TT‐NH_2_	[Ac‐Lys1346;Asn(Glc)1352]HMW1(1346–1354)‐NH_2_	Peptide 1’_B_
Ac‐KAN(Glc)VTLN(Glc)TT‐NH_2_	[Ac‐Lys1346; Asn(Glc)1348;Asn(Glc)1352]HMW1ct(1346–1354)‐NH_2_	Peptide 1’_A,B_
Ac‐KAN(Glc)VTLN(Glc)TTG‐K(N3)‐NH_2_	[Ac‐Lys1346;Asn(Glc)1348;Asn(Glc)1352;Lys(N3)1356]HMW1ct(1346–1356)‐NH_2_	Peptide 1′_A,B_GK(ϵN_3_)

### Dextran functionalization

Dextran is a versatile biopolymeric scaffold that can be functionalized with several reactive moieties to link different types of biologically active cargoes, i. e., peptides, proteins, cyclodextrins, and small molecules.[^19^, ^20^, ^21^]

Dextran is composed of α‐1,6 glucose units. This bond (with the primary alcohol of glucose) gives great flexibility to the polysaccharide chain even in large molecules. This particular characteristic induces unstructured conformation and gives impressive solubility in water, compared to polysaccharides of the same size but other linkages. Dextran is therefore well suited to act as an active cargo. In particular, 40 KDa dextran has an average degree of polymerization of 250 (40000/162) and taking into account 4.6 Å per glucosyl moiety (C1–O6 distance), the maximum length range is approximately 1150 Å. This length can be considered large enough to cover many/all IgM paratopes.

We selected the CuAAC reaction to generate in the biopolymer a multivalent presentation of N‐Glc moieties, fundamental for antibody binding in Multiple Sclerosis. In particular, the azide‐containing diglucosylated Peptide 1’N_3_ was grafted to the glycidyl‐propargyl functionalized dextran (Dex40‐GP). Dex40‐GP was obtained by functionalization with alkynyl moieties by an epoxide opening reaction of glycidyl propargyl ether (GPE) in aqueous basic solution, according to a previously described procedure.^[20]^ The glycidyl linker provides a suitable distance between the grafted peptides and dextran without affecting solubility. Moreover, the ether bond is hydrolytically stable. Basic water solution is used as a solvent to avoid homopolymerization of GPE, which is used in excess to overcome a competing hydrolysis.

**Scheme 1:**
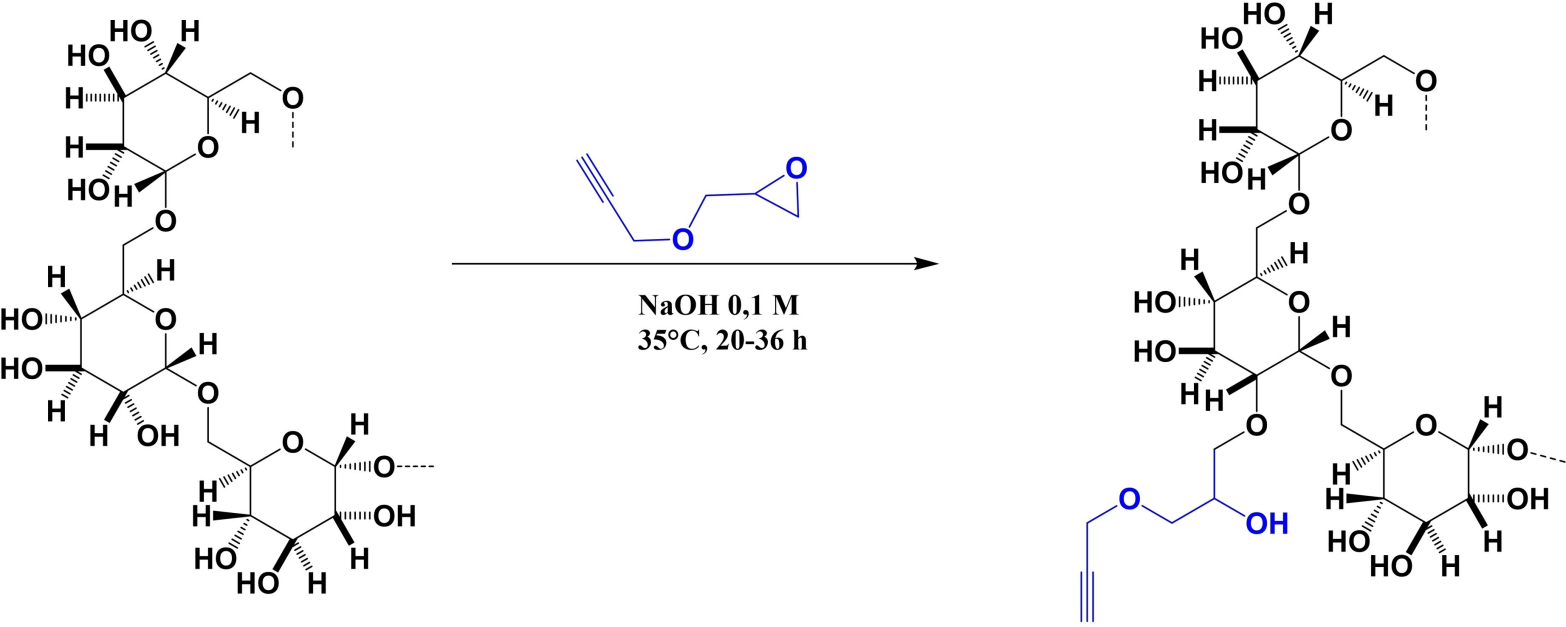
Functionalization of 40 kDa dextran with glycidyl‐propargyl moieties.

Approximating dextran as a linear polymer composed of α‐1,6 linked glucose units, GPE is possibly reacting with one out of the three free hydroxyl functions (O2, O3, O4). The precise hydroxyl function involved in the new ether bond formation is the object of an open debate.[^20^, ^22^, ^23^] The less hindered position is O3, however the H1 chemical shift of the newly modified units likely corresponds to O2/O4 functionalized glucose. The hydroxyl at position 2 of the glucose unit is generally shown to be the most substituted in these conditions. It is the most acidic, and thus more deprotonated by sodium hydroxide and more alkylated.^[24]^ Nevertheless, for simplicity's sake, the modified dextran is always represented as a homogeneously O2‐modified construct (Scheme 1).

**Figure 1:**
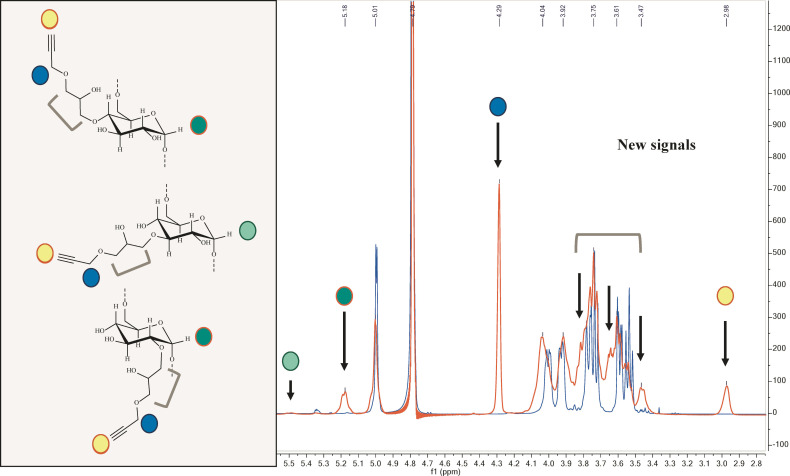
^1^H‐NMR overlaid spectra of the commercially available 40 kDa dextran (blue spectrum) and GPE functionalized Dex40‐GP (red spectrum): new signals appearance that were assigned to the corresponding protons of the GP‐functionalized glucose units are indicated by colored circles.

The degree of substitution (DS) on the hydroxyl groups of dextran was calculated by q^1^H‐NMR analysis. The percentage of propargyl moieties per glucose unit in dextran were calculated as the integral ratio of alkyne proton (2.98 ppm) and anomeric protons (5.60‐4.95 ppm range) resulting 29 % (see Supporting Information for details). The DS of the modified dextran units is 0.31, calculated as ratio of the new H1 signals of modified glucoses and total anomeric protons area. Moreover, the area of the signal at 4.29 ppm (methylene protons of propargyl group) and the total area of peaks between 4.15–3.35 ppm are both higher than expected (Figure 1). This unreported outcome was ascribed to the minor presence of GPE oligomers and alkyne‐alkyne coupling (Scheme S1 in the Supporting Information). The average oligomerization degree, calculated by the ratio between the signal at 4.28 ppm due to the CH_2_ of all propargyl moieties and the signal of the anomeric protons of the functionalized glucose units in dextran was 1.8. According to our calculations, out of the 246.9 glucose units in each dextran molecules, 170.4 units remain as simple α‐1.6 glucoses (MW=162 Da) and 76.5 units are α‐1,6 glucoses modified with 1.8 GP groups (MW=366 Da) each on average. Among these, 71.6 terminal alkyne protons are present. In conclusion, the estimated molecular weight of Dex40‐GP is 55.2 kDa (see Supporting Information, section 2.3.2).

### Conjugation of Dex40‐GP to Peptide 1’N_3_

**Scheme 2:**
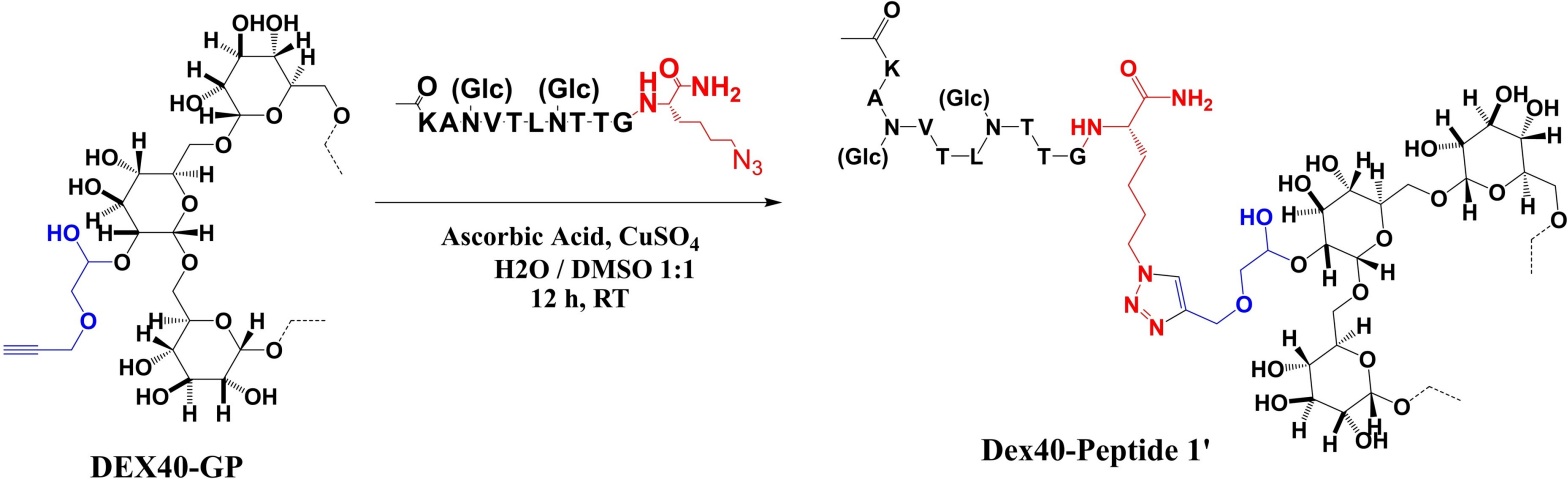
CuAAC promoted reaction between the diglucosylated peptide 1′_A,B_GK(ϵN_3_) and Dex40‐GP, leading to the multivalent peptide‐dextran conjugate Dex40‐Peptide 1.

Peptide 1′_A,B_GK(ϵN_3_) was used for conjugation to Dex40‐GP by CuAAC in a DMSO/water mixture (Scheme 2).

**Figure 2:**
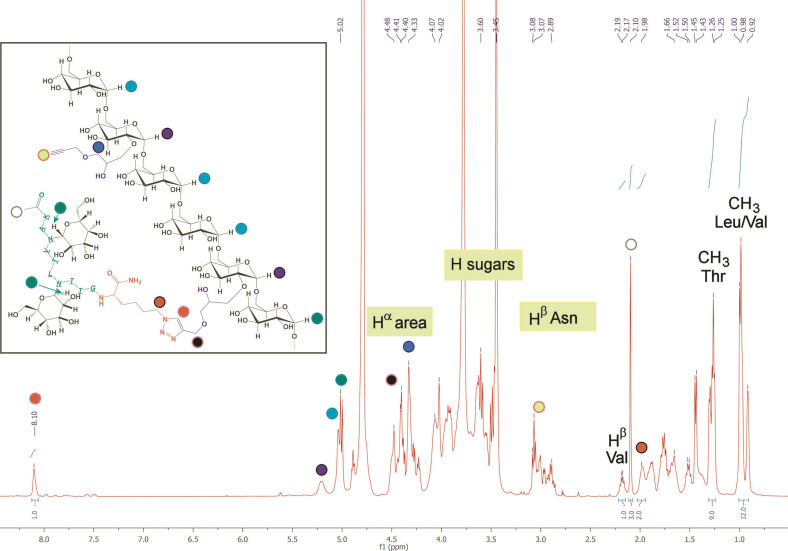
^1^H‐NMR of Dex40‐Peptide 1’. Signals and areas assignments characterizing the novel multivalent peptide‐dextran conjugate are reported in colored circles.

After a thorough work‐up for copper removal, the obtained Dex40‐Peptide 1’ was characterized by NMR spectroscopy. To facilitate peaks assignment and spectra interpretation, ^1^H and ^13^C‐NMR of peptide 1′_A,B_GK(ϵN_3_) were also recorded (see Experimental Section for further information). The characteristic peak at 8.10 ppm in the ^1^H‐NMR spectrum of Dex40‐Peptide 1’ provides clear evidence of the presence of 1,4‐disubstituted 1,2,3‐triazole ring as amide bond surrogate, linking Dex40 to Peptide 1’ (Figure 2). The integral value of the proton signal (8.10 ppm) of the formed triazolyl moiety is in the expected ratio with other isolated signals originating from Peptide 1′_A,B_GK(ϵN_3_) protons, such as H^β^ of valine (2.18 ppm), methyl protons of the N‐terminal acetamide, as well as characteristic signals of threonine, leucine, and valine residues thus indicating that the intact peptide is covalently linked to the dextran backbone (Figure 2). This evidence is important to prove that no peptide in the sample is simply absorbed by the polymer.

Peptide 1 linking through triazole bridges is also evidenced by the shift of H^ϵ^ signal of Lys(N_3_) from 3.35 ppm to 1.98 ppm. Instead, the signal due to methylene protons of propargyl group (4.29 ppm) decreases in intensity without disappearing, while the new signal of triazole‐linked methylene appears at 4.50 ppm. Terminal alkynes signal (2.98 ppm) also decreases without disappearing completely, as expected (see the Experimental Section for further information). Most importantly, using the isolated triazole proton as reference signal, the degree of substitution of dextran in peptide molecules was calculated by comparing it with integrals of relevant peaks/ranges, such as H^1^ proton of modified dextran units. The peptide loading in dextran was calculated to be ∼0.20 peptide mol/glucoside units, meaning that 31 % mol of terminal alkynes initially present on dextran remain unreacted (residual alkynes on dextran DS=0.1) and can be exploited for future applications (see Experimental Section for further information). According to these considerations, each molecule of Dex40‐Peptide 1’ carries on average 48 peptide branches and thus it has a final estimated MW of ≈129 kDa (Table 2).

**Table 2:** Estimated molecular weights (MW) and units of compounds DEX40, DEX40‐GP, and DEX40‐Peptide 1’.

Compound	Estimated MW	Estimated units
DEX40	40 KDa	247 α‐1,6‐Glc units/molecule
DEX40‐GP	55 KDa	170 unmodified α‐1,6‐Glc units/molecule 77 GP‐linked α‐1,6‐Glc units/molecule (31 %) 1.8 GP groups/unit 72 terminal reactive alkyne groups/molecule (29 %)
Dex40‐Peptide 1’	139 KDa	170 unmodified α‐1,6‐Glc units/molecule 24 reactive alkynes/molecule (9.5 %) 48 peptides/molecule (19.5 %)

**Figure 3:**
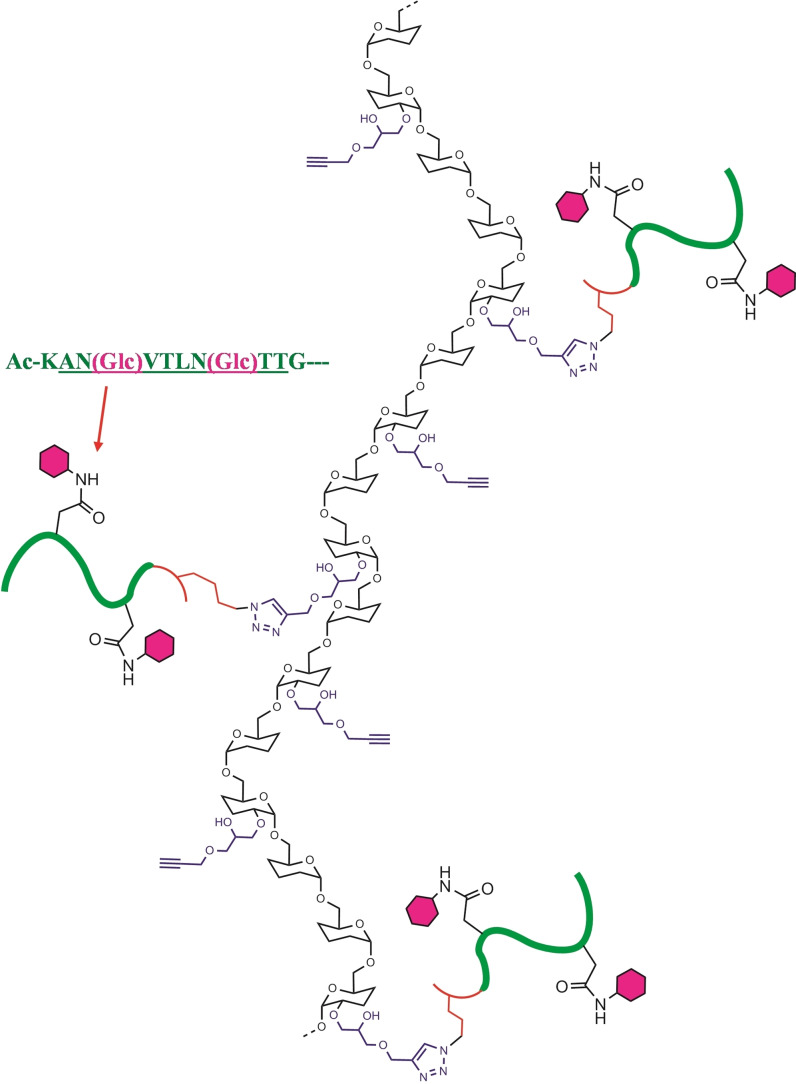
Schematic representation of the novel conjugate Dex40‐Peptide 1’, carrying HMW1ct(1347–1354) diglucosylated epitopes shared by Peptide 1’_A,B_. On the right, main features of dextran and dextran derivatives, based on NMR characterization, are summarized.

The estimated glucose/peptide ratio of 5/1 lead to a calculated distance between antigens (in an idealized extended dextran with regular antigen space) of 23 Å (4.6×5). Although not easy to go further in conjugate characterization, we expect a good complementarity of both macromolecules. Since the distance of paratopes is ca 120 Å in one monomeric immunoglobulin molecule, these conjugates are thus potentially able to link several paratopes at the same time, acting as tentacles able to trap antibodies (Figure 3).

### Application of multivalent peptide‐dextran conjugate to antibody detection and capture

To assess the ability of the novel peptide‐dextran conjugate Dex40‐Peptide 1’ to identify anti‐hyperglucosylated adhesin antibodies in MS patient sera, we performed competitive ELISA experiments. To this purpose, five representative patient sera were selected based on their anti‐HMW1ct(N‐Glc) antibody titers. MS1 and MS2 sample sera presented high IgG and low IgM titer. Conversely, MS4 and MS5 sample sera showed lower IgG titer compared to IgM one. Finally, MS6 sample serum was selected because of its high titer of both IgG and IgM antibodies.

### Competitive ELISA

The aim of this experiment was to compare the affinities of IgG and IgM antibodies to the new multivalent peptide‐dextran conjugate Dex40‐Peptide 1’ with those toward the parent hyperglucosylated adhesin protein. Therefore we performed competitive ELISA using HMW1ct(N‐Glc) as coating antigen and measuring the ability of Dex40‐Peptide 1’, added in solution, to inhibit IgG and IgM binding to the coated antigen. In addition, we also tested the starting materials based on dextran, Dex40, its alkyne‐functionalized analogue, Dex40‐GP, and, only for IgM, Peptide 1’_AB_ and unglucosylated adhesin, HMW1ct.

**Figure 4:**
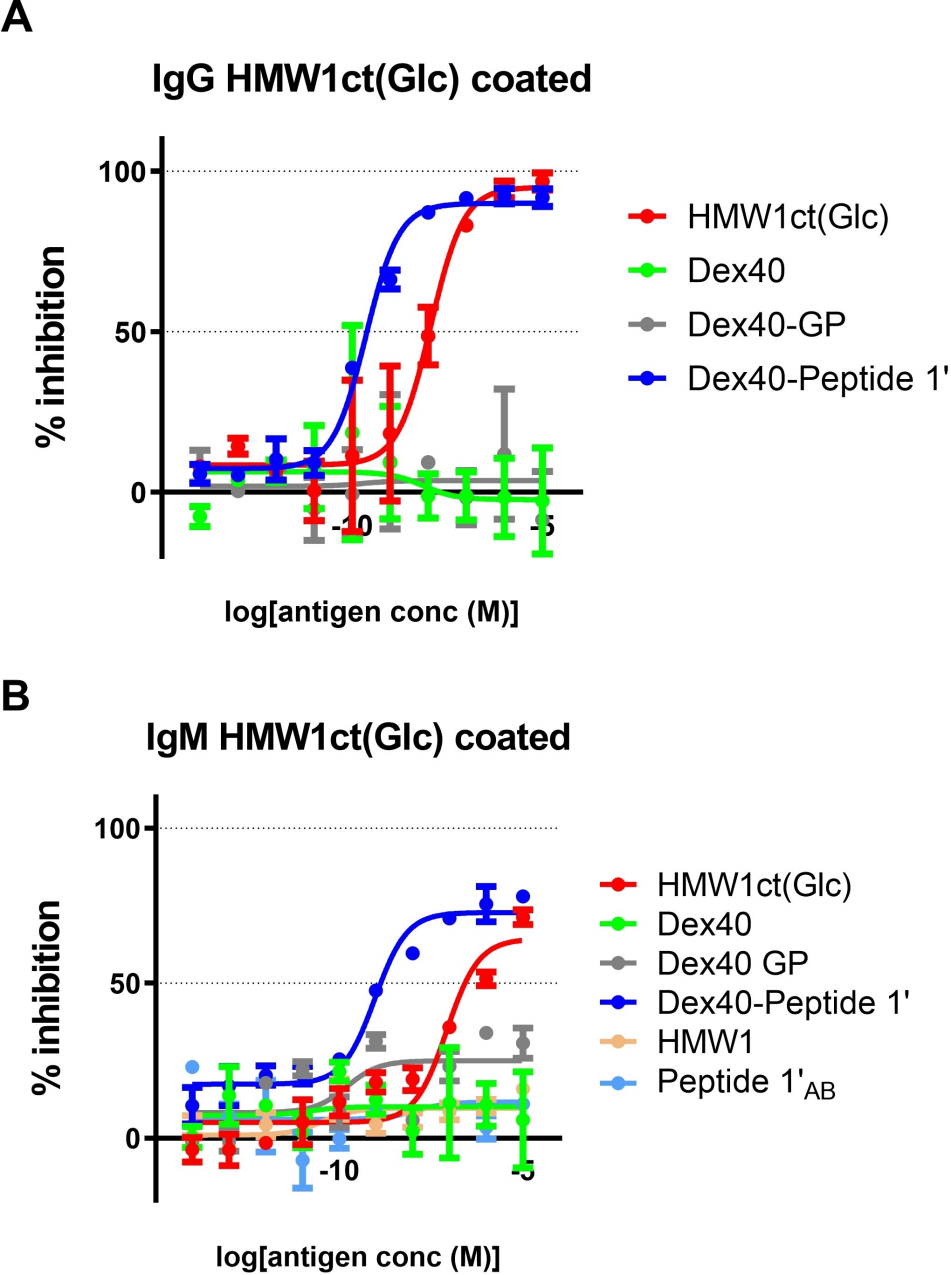
Competitive ELISA obtained coating the hyperglucosylated protein antigen HMW1ct(N‐Glc). Inhibition curves of anti‐HMW1ct(N‐Glc) IgG (A) and IgM (B) antibodies using Dex40, its alkyne‐functionalized analogue Dex40‐GP, and the novel peptide‐dextran conjugate Dex40‐Peptide 1’. Results show the inhibition activity % (ordinate axis) of a representative serum MS1 for IgG (A) and of a representative serum MS4 for IgM (B) vs antigen concentrations on a logarithmic scale (abscissas axis).

The results, showed in Figure 4, clearly indicate that anti‐HMW1ct(N‐Glc) antibody affinity toward Dex40‐Peptide 1’ is higher than that toward the hyperglucosylated protein. In particular, the multivalent peptide‐dextran conjugate Dex40‐Peptide 1’ displayed an IC_50_ 1.56 and 1.96 log units lower for IgGs and IgMs, respectively (Table 3). At variance, the starting polymer, Dex40, and its alkyne‐functionalized analogue, Dex40‐GP, do not compete for antibody binding. Therefore, antibody recognition can be ascribed only to the presence of Peptide **1’** linked to Dex40.

**Table 3:** Calculated pIC_50_ values of anti‐HMW1ct(N‐Glc) IgG and IgM antibodies to HMW1ct(N‐Glc) and Dex40‐Peptide 1’. Values are reported as 95 % confidence interval for the calculated mean pIC50±the standard error (SEM) in representative sera MS1 (IgG) and MS4 (IgM).

Antigen	pIC_50_ (IgG)	pIC_50_ (IgM)
HMW1ct(Glc)	8.14±0.45	7.07±0.72
Dex40‐Peptide 1’	9.70±0.21	9.03±0.39

This is the first insight that a tentacle‐like macromolecule decorated with multiple copies of the selected N‐glucosylated antigen Peptide **1’_A,B_** is able to bind anti‐bacterial hyperglucosylated adhesin IgGs and, most importantly, IgMs from MS patient sera. Particularly in the case of IgMs, the differences in IC_50_ can be ascribed to an increased avidity because of a multivalent effect (Table 3). This hypothesis is strongly supported by the observation that the diglucosylated Peptide **1’_A,B_**_,_ containing the same epitope, lacks multivalent presentation of N‐glucosyl moieties. It is worth to note that Peptide **1’_A,B_** is able to compete with a structure‐based designed monovalent antigenic probe,^[15]^ but not to inhibit anti‐HMW1ct(N‐Glc) IgM antibodies (Figure 4, panel B).

### Solid‐phase ELISA

Antibody detection in patient sera using the tentacle‐like polymer Dex40‐Peptide 1’ has also been evaluated in solid‐phase ELISA (SP‐ELISA). At this purpose, preliminary tests were carried out to set‐up the optimal conditions for an SP‐ELISA screening of a larger collection of sera. Dex40‐Peptide 1’ coated on polystyrene ELISA plate efficiently detected both IgG and IgM antibodies. Since we have never observed a linear correlation between the antigen concentration in the coating solution and the observed signal, we coated both the peptide‐dextran conjugate and the glucopeptide at the same formal concentration.

**Figure 5:**
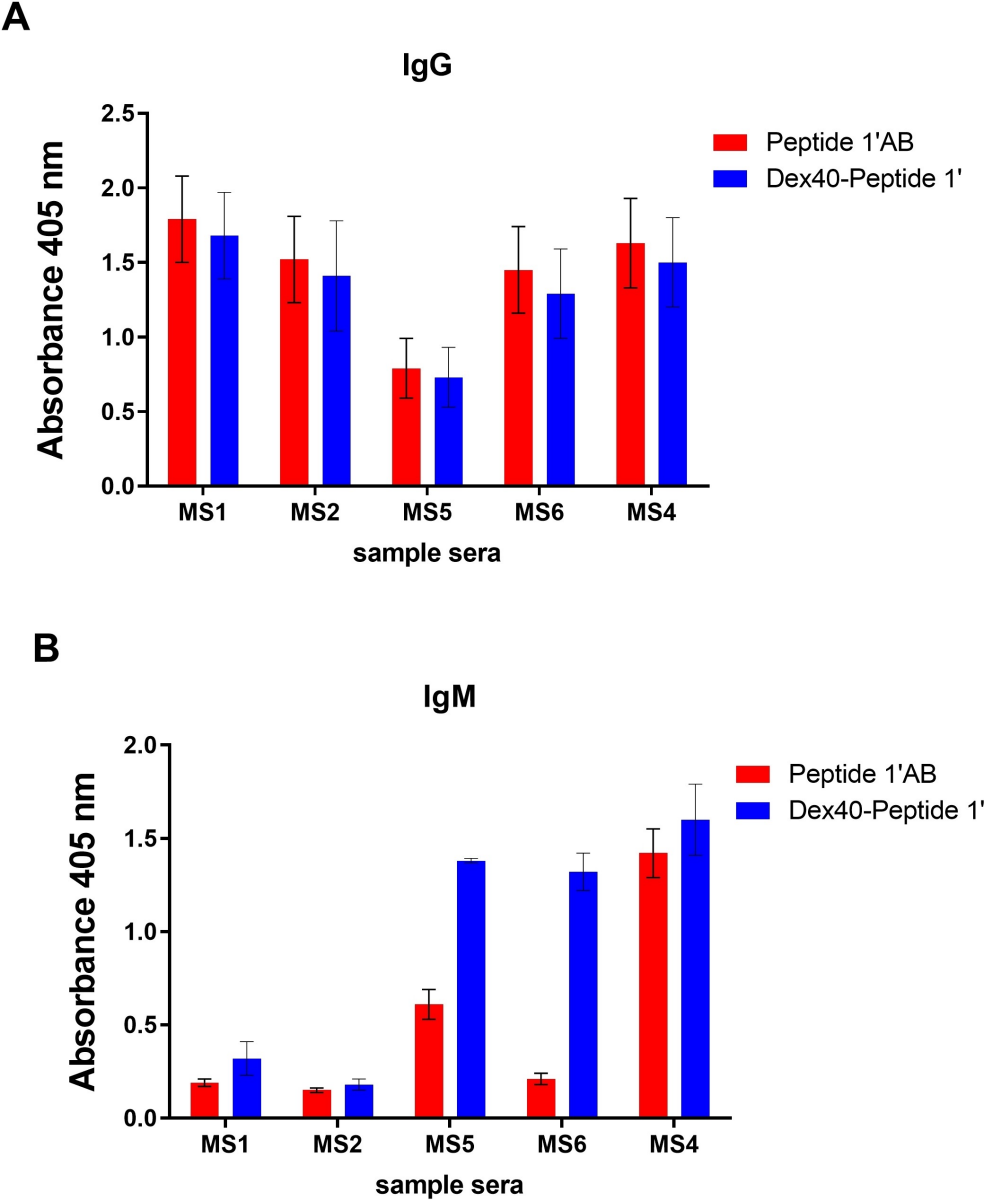
SP‐ELISA to detect IgG (A) and IgM (B) antibody titers in 5 representative Multiple Sclerosis sera samples using the diglucosylated Peptide 1’_AB_ (red) and Dex40‐Peptide 1’ (blue) as antigens. Mean absorbance values were evaluated at 405 nm; standard deviation (SD) for n=3 independent measures evaluated at the same conditions are reported as error bars.

In the case of IgGs (Figure 5, panel A), similar antibody titers are observed using as coating antigen either the polymer or the diglucosylated Peptide 1’_A,B_. These data indicate that when the polymer is coated on the plate to detect IgGs, the multivalency is not an added value. In fact, the peptide‐bearing polymer and the free peptide behave similarly as coating antigens. At variance, when measuring IgMs in the three representative sera in which the IgM isotype is relevant, i. e., MS3, MS4, and MS5, Dex40‐Peptide 1’ was able to identify higher IgM antibody titers, as compared to the free glucopeptide (Figure 5, panel B). Therefore, IgM antibody identification significantly benefits of the tentacle‐like structure of Dex40‐Peptide 1, presenting multiple copies of Peptide 1’_A,B_. This result is in agreement with our previous observation that short N‐glucosylated sequences were able to detect IgG‐type antibodies, but clearly displayed a drop in IgM antibody recognition.^[25]^

### Antibody purification

The novel tentacle‐like antigen Dex40‐Peptide 1’ was therefore tested in a proof‐of‐concept experiment of specific antibody purification from MS sample sera.

Firstly, we immobilized Dex40‐Peptide 1’ onto CNBr‐preactivated Sepharose resin beads, in order to set up the stationary phase for an immunoaffinity‐based column. In this conjugation step, the amine functions on side‐chain lysine residues react with the activated cyanate active ester on the Sepharose resin forming a stable isourea derivative. A solution of Dex40‐Peptide 1’ (1 mg/mL) was incubated with 100 mg of Sepharose resin beads in the coupling buffer (NaHCO_3_ 0.1 M, NaCl 0.5 M at pH 8.3). Each diglucosylated peptide sequence (Ac‐KAN(Glc)VTLN(Glc)TT) grafted onto the dextran biopolymeric scaffold (bearing a total of 48 peptides according to the NMR analysis, Figure 2), contains an N‐terminal lysine residue. Therefore, ca. 0.4 μmol of primary amine function/mg of tentacle are available to link to Sepharose. Immobilization was verified by comparing UV spectra of the initial solution of Dex40‐Peptide 1’ with the eluted solution after incubation. Although analysis of the Dex40‐Peptide 1’ initial solution before coupling did not display a clear maximum intensity peak, the absorbance resulting after coupling dropped to zero. This allowed us to consider that the reaction was completed.

Unreacted cyanate sites were blocked with a glycine solution and functionalized Sepharose beads were extensively washed and equilibrated. Diluted serum sample MS5 (FT1), selected for its high IgM antibody titer, was flowed through the functionalized Sepharose. Then, final flow‐through was collected (FT2).

**Figure 6:**
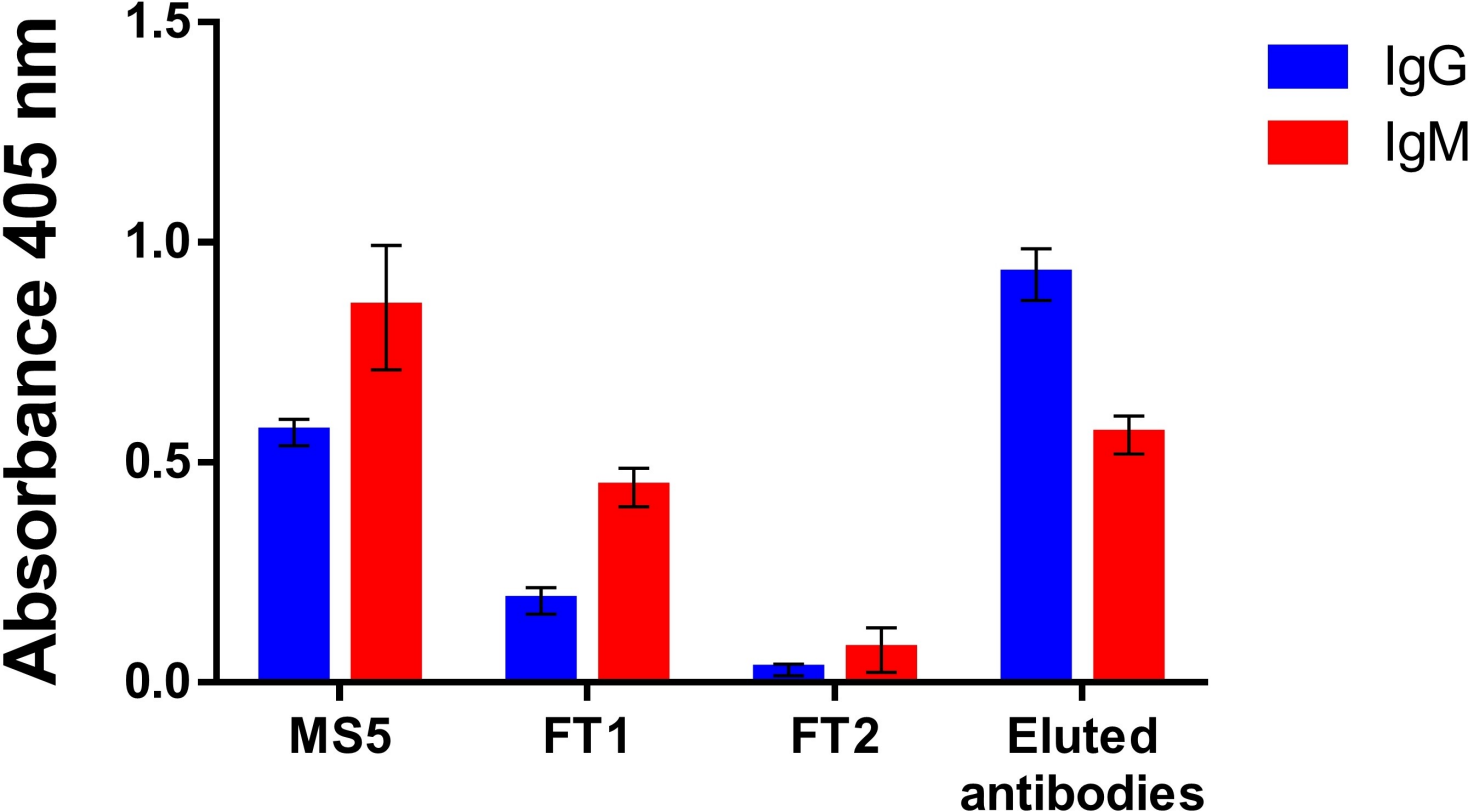
SP‐ELISA to detect IgG and IgM antibody titers after immunoaffinity purification of representative MS5 sample serum, on Sepharose column functionalized with Dex40‐Peptide 1’. The multivalent peptide‐dextran conjugate Dex40‐Peptide 1’ was coated on the ELISA plate. Mean absorbances (±standard deviation for n=3 independent experiments) of IgG and IgM antibodies of the different fractions are plotted. MS5 (sample serum), FT1 (diluted sample serum), and FT2 (final flow‐through) were tested using same dilution ratio. Eluted antibodies were approximately 5 times more concentrated.

Retained antibodies were finally eluted from the column using acidic glycine solution (pH=2.3), which was immediately neutralized after elution, by addition of NaHCO_3_ buffer. The eluted solution containing the purified antibodies was exchanged with PBS and isotype antibody titer was evaluated by SP‐ELISA (Figure 6).

Results showed that FT2 fractions displayed a remarkable decrease in antibody titer indicating that both IgG and IgM‐type antibodies were almost completely depleted.

The slight decrease in IgM antibody titer in the eluted antibody fraction compared to the original titer in the initial MS5 sample serum, can be ascribed to lower stability of pentameric IgMs in the acidic conditions of the eluent that is required for eluting the antibodies from the Sepharose matrix. Moreover, residual IgMs have possibly remained strongly linked to Sepharose, because of the high affinity binding of IgMs to Dex40‐Peptide 1’.

However, our results demonstrate that specific MS antibodies to bacterial diglucosylated adhesin peptide of non‐typeable *Haemophilus influenzae*, particularly of the IgM isotype, can be selectively captured by an immunoaffinity procedure based on the novel multivalent dextran conjugate Dex40‐Peptide 1’, bearing multiple copies of the relevant bacterial peptide epitope.

## Conclusion

We reported here the synthesis and characterization of a new dextran‐peptide conjugate that holds great potential for future theragnostic and prognostic applications. The assembly of multiple N‐glucosylated peptide moieties grafted to a polymer backbone is crucial for the detection and capture of IgG and, most importantly, IgM antibodies in sera of MS patients. Peptide 1′_A,B_GK(ϵN_3_), containing the diglucosylated HMW1ct(1347–1354) epitope, was grafted onto an alkyne‐modified dextran by CuAAC promoted coupling (click chemistry). Quantitative NMR characterization allowed to measure the peptide loading of the polymer as 48 branches /mol per dextran chain. This novel grafted polymeric structure was shown to dramatically increase the binding capacity of IgGs and IgMs in MS sera. The effect of cooperativity resulted in an increase in antigenicity when compared to the free epitope by competitive ELISA tests. The natural evolution of this project points toward the exploitation of this synthetic polymer for the isolation and characterization of antibodies. In fact, a representative MS serum was successfully depleted from IgG and IgM antibodies that recognize diglycosylated HMW1ct antigen conjugated to a Sepharose column, as confirmed by SP‐ELISA. This preliminary achievement is a proof‐of‐concept of the selective capture of circulating autoantibodies that could hopefully lead to the development of a specific apheresis‐based device. Moreover, these results pave the way for future elaborations involving the production and characterization of new conjugates with different peptide loading (i. e., the degree of substitution) and polymer size (i. e., dextran molecular weight). Finally, it is important to note that CuAAC reaction between Peptide 1′_A,B_GK(ϵN_3_) and Dex40‐GP was intentionally driven to preserve a minor percentage of unreacted alkynes that could be useful for future applications. The use of 0.8 eq of azide (i. e., peptide)/alkyne group, results of Dex40‐Peptide 1’ containing 19.5 peptide chains and 9.5 terminal alkynes/molecule. Each peptide sequence contains a N‐terminal lysine carrying a primary ϵ‐amino function useful for bioconjugation purposes. In our preliminary attempts, we exploited the primary amine groups for the immobilization of Dex40‐Peptide 1’ to CNBr‐activated Sepharose beads. This approach allowed us to verify the efficacy of our conjugate for antibody removal from a patient serum sample. However, the reactivity of alkyne‐terminal groups is not restricted to the development of stationary phases for immunoaffinity purification. Several bioconjugation strategies can take advantage of the orthogonality between the alkyne and the amino groups. Indeed, the reaction with other molecules could in principle provide multifunctional architectures. For example, we can envisage the use of lipids for immunostimulation,^[26]^ thiol containing molecules for gold nanoparticle‐based delivery^[27]^ or gold surface functionalization in sensor chips,[^28^, ^29^] as well as biotin, maleimide and many other functionally reactive derivatives that can be exploited for the detection and characterization of anti‐N(Glc) antibodies. In summary, Dex40‐Peptide 1’, containing unreacted alkynes and the primary ϵ‐amino function on the N‐terminal lysyl residue in the conjugated Peptide 1′_A,B_, is the relevant component of a technology platform that may enable the construction of more elaborate molecular systems amenable to new and exciting applications in chemical immunology.

## Conflict of interest

The authors declare no conflict of interest.

## Supporting information

As a service to our authors and readers, this journal provides supporting information supplied by the authors. Such materials are peer reviewed and may be re‐organized for online delivery, but are not copy‐edited or typeset. Technical support issues arising from supporting information (other than missing files) should be addressed to the authors.

Supporting Information
