# Supplementary material for: Selective Capture of Anti‐N‐glucosylated NTHi Adhesin Peptide Antibodies by a Multivalent Dextran Conjugate
Source: Chembiochem. 2021 Dec 6;23(3):e202100515. doi: 10.1002/cbic.202100515 (PMC9300045; doi:10.1002/cbic.202100515)
Supplement: Supplementary file 1 — Supporting Information [file CBIC-23-0-s001.pdf]

ChemBioChem

Supporting Information

Selective Capture of Anti-*N*-glucosylated *NTHi* Adhesin Peptide Antibodies by a Multivalent Dextran Conjugate

Antonio Mazzoleni, Feliciana Real-Fernandez, Francesca Nuti, Roberta Lanzillo, Vincenzo Brescia Morra, Paolo Dambruoso, Monica Bertoldo, Paolo Rovero, Jean-Maurice Mallet, and Anna Maria Papini*

Table of Contents

1. Peptides synthesis
2. Dextran conjugates
3. Protein Expression
4. ELISA
5. Immunoaffinity column

1. PEPTIDE SYNTHESIS

Peptide synthesis was performed on a manual batch synthesizer (PLS 4×4, Advanced ChemTech) by Fmoc-based solid phase strategy in fritted syringes. Generally, peptide chains were assembled by sequential coupling of activated Fmoc-amino acid (5 equiv) in DMF (1 mL x 100 mg resin) in the presence of HBTU (4.5-5 equiv) and DIEA (7-10 equiv) for 20 min at room temperature. Resins were then washed with DMF (x2) and CH_2Cl_2 (x2) and the completeness of each coupling was ascertained by the Kaiser's test. Fmoc deprotection was carried out twice (5 min, 10 min) by treatment with piperidine (20% v/v in DMF) followed by washing with DMF, DCM, and DMF. Fmoc-Asn[Glc(OAc$_4$)]-OH, Fmoc-Lys(N$_3$)-OH and Fmoc-Pra-OH couplings (2.5 eq) were performed in DMF (1 mL x 100 mg resin) in the presence of HATU (2.5 equiv) and DIEA (5 equiv) for 60 min at room temperature. N-terminus acetylation reactions were performed by double treatment for 10 min in DMF/Ac_2O (4:2), 1 mL/100 mg resin. After complete elongation of the peptide chains, the resins were washed with DCM, then methanol, and finally dried.
The final cleavage from the resin and side-chain deprotection were performed by treatment for 3 h with TFA/TIS/H2O (95:2.5:2.5), 1 mL/ 100 mg resin. Precipitation in diethyl ether at 4°C and lyophilization afforded crude peptides.
The removal of the acetyl protecting groups of the β-D-glucopyranosyl moiety linked to the asparagine residue side chain was carried out by treatment with 0.05 M MeONa in dry MeOH (pH 12, 5-10 mg lyophilized crude/mL solution, 2-3 h)[1]. The reaction was quenched by adding conc. HCl to neutrality, the solvent was evaporated under vacuum and the residue lyophilized.
All peptides were purified through semipreparative RP-HPLC (>95%) to be used for autoantibody detection and characterized through RP-HPLC and mass spectrometry using methods and solvent system reported in tables. Peptides were purified by RP-HPLC on a ACE 5 C18-300" (250 × 10 mm) column (Waters, Saint Quentin en Yvelines, France) at 28°C using a Waters instrument (Sepation Module 2695, detector diode array 2996) working at 10 mL/min, with the indicated linear gradients. The solvent systems used were: A (0.1% TFA in H2O) and B (0.1 % TFA in MeCN). Characterization was performed by RP-HPLC (UltiMate, Thermo Scientific) equipped with a ACE 5 C18-300" (250 × 4.6 mm) column working at 1 mL/min, with UV detection at 220 and 280 nm. The solvent systems used were: A (0.1% TFA in H2O) and B (0.1 % TFA in MeCN). Mass spectral analysis was performed by MALDI-TOF (Voyager-DETM PRO Workstation, Applied Biosystems) in positive ion reflector mode using the matrix α-Cyano-4-hydroxycinnamic acid (CHCA). The products were lyophilized with an Edwards apparatus, model Modulyo. Analytical data of peptides are summarized in Table S1.

Table S1 – Analytical data of peptides

Compound	Purification Method	t_r (min)[a]	m/z	Mass (mg)	Yield
**Peptide 1**	5-40 % B, 10 mL/min in 10 min	6.32[b]	1002.28 [1002.55]	36	66%
**Peptide 1'$_A$**	10-30 % B, 10 mL/min in 10 min	5.80[b]	1186.18 [1164.60]	14	22%
**Peptide 1'$_B$**	10-30 % B, 10 mL/min in 10 min	5.95[b]	1326.35 [1326.66]	17	24%
**Peptide 1'$_{A,B}$**	10-30 % B, 10 mL/min in 10 min	5.90[b]	1164.37 [1164.60]	23	36%
**Peptide 1'$_{A,B}$GK(εN$_3$)**	20-35 % MeCN, 4 mL/min in 30 min	3.90[b]	1538.2 [1537.8]	22	13%

[a]Analytical RP-HPLC gradients at 0.6 mL min^{-1} in 5 min; solvent system A: 0.1% TFA in H_2O, B: 0.1% TFA in MeCN. [b] 10-60% B in 5 min.

2. DEXTRAN CONJUGATES

2.1 Synthesis of Dex40-GP

To a stirring solution of dextran 40 kDa (1 g, 25 µmol dextran, 6.2 mmol glucose units) in 0.1 M aq. NaOH (7 mL) was added glycidyl propargyl ether (1 mL, 9.3 mmol). The mixture was stirred at 35°C for 20 h then added dropwise in isopropyl alcohol (100 mL). The white precipitated compound was thoroughly filtered through a sintered glass filter and washed with 1-propanol. The crude solid was dissolved in water (≈ 9 mL) and dialyzed in a cassette (Slide-A-Lyzer 10K MWCO, Thermo Fisher Scientific) against Milli-Q water until the conductivity of the solution was ≈ 0 (48 h). The alkyne dextran (1.103 g, Final estimated MW ≈ 55 kDa, 80% recovery yield) was obtained as a white compact powder after lyophilization. The degree of substitution (DS) of dextran as determined by ^{1}H-NMR was 0.29 as glycidil ether moieties for glucosidic repeats.

2.2 Synthesis of Dex40-Peptide 1’

To a stirring solution of Dex40-GP (10 mg, 13.0 µmol propargyl groups, 1 eq) and Peptide $1'_{A,B}GK(\varepsilon N_3)$ (15 mg, 9.8 µmol, 0.75 eq) in DMSO (800 µL) and water (500 µL), a mixture of 100 µL of $CuSO_4$ 1M (5 eq) and 200 µL Ascorbic acid 1M (10 eq) was added.
The pale-orange solution was left under stirring overnight. Then 20 mL of a solution EDTA 10 mM were added and the solution was lyophilized. The dried crude was dissolved in 15 mL H_2O Milli-Q and centrifuged with Amicon Ultra Centrifugal Filters (MWCO = 30 kDa), then buffer-exchange steps with H_2O Milli-Q were performed to remove all EDTA (final washing volume ≈100 mL, Dex40-Pept6 ≈1 mL in H_2O). After lyophilization the desired product is obtained as a white powder (23 mg, degree of substitution = 0.19 (e.g. 1 peptide chain / 5 glucose units). Final estimated MW ≈ 130 kDa, 89% yield). The degree of substitution (DS) was determined by ^{1}H-NMR.

2.3 NMR analysis of dextran-based conjugates

NMR spectra of Dex40, Dex40-GP, Peptide $1'_{A,B}GK(\varepsilon N_3)$ and Dex40-Peptide 1’ were acquired in D_2O (15-25 mg/mL) with a Bruker 500 MHz spectrometer. Preliminary inversion recovery experiments were conducted for each sample in order to find the maximum longitudinal relaxation time (T1) and set the appropriate delay time (d1) for quantitative ^{1}H-NMR analyses. ^{1}H spectra were calibrated using the residual water signal in accordance with literature[2]. ^{13}C chemical shifts were calibrated indirectly based on the ^{1}H calibration.

2.3.1 NMR of Dextran 40 kDa (Dex40)

NMR spectra of Dex40 present the main peaks originating from protons and carbons of the α-1,6 linked glucoses (most abundant form). Other minor peaks are assigned (Figure S1).
^{1}H NMR (500 MHz, D_2O) δ 5.00 (d, *J* = 3.6 Hz, 1H, H^1), 4.01 (dd, *J* = 11.5, 4.6 Hz, 1H, H^6), 3.93 (ddd, *J* = 10.2, 4.5, 2.2 Hz, 1H, H^5), 3.77 (*pseudo*-d, *J* = 11.5 Hz, 1H, $H^{6'}$), 3.74 (*pseudo*-t, *J* = 10.0 Hz, 1H, H^4), 3.59 (dd, *J* = 9.9, 3.7 Hz, 1H, H^2), 3.54 (*pseudo*-t, *J* = 9.5 Hz, 1H, H^3).
^{13}C NMR (126 MHz, D_2O) δ 100.5 (C1), 76.2 (C3), 74.2 (C2), 73.0 (C4), 72.3 (C5), 68.4 (C6) ppm.

2.3.2 NMR of Dex40-GP

NMR spectra of Dex40-GP show the success of alkyne functionalization of dextran molecules. Proton spectrum was used to characterize the novel construct, which was found to carry a minor component of oligomerized and alkyne-alkyne coupled GP groups (Scheme S1).
Considering the area of H1 signals as 100 (total glucose units) (Figure S2):

The degree of substitution (DS) was calculated as follows:

- Percentage of O-modified glucose units/glucose units in dextran:
  $\frac{(A_1-A_2)\times 100}{A_1} = \frac{(100-69.1)\times 100}{100} = 30.9 \approx 31\%$

  A_1 = Area of the anomeric proton signals at 4.95-5.55 ppm (normalized at 100%)

  A_2 = Area of the anomeric proton signal of unfunctionalized glucose units at 5.01 ppm (Figure S2).
- Percentage of reactive alkynyl functions/glucose units in dextran:
  $\frac{A_3\times 100}{A_1} = \frac{28.7\times 100}{100} = 28.7 \approx 29\%$

  A_1 = Area of the anomeric proton signals at 4.95-5.55 ppm (normalized at 100%)

  A_3 = Area of the proton signal of the terminal alkynyl functions at 2.98 ppm (Figure S2)
- Average number of GP functions/glucose-GP unit:

  $$\frac{(A_4/2)}{A_1 - A_2} = \frac{(109.6/2)}{100 - 69.1} \approx 1.8$$

  A_4 = Area of the 2 protons of the CH_2-α- in any alkynyl function at 4.28 ppm

  $(A_1 - A_2)$ = Area of the anomeric protons of the functionalised glucose units in Dex40-GP (Figure S2)

These calculations can be used to estimate the expected area of signals in 4.15 - 3.38 ppm range (A_5): 6 × (69.1 + 30.9) + 5 × 30.9 × 1.77 = 873.5. This value is in accordance with the observed one.
^{1}H NMR (500 MHz, D_2O) δ 5.52 – 4.95 ($\underline{H}^1$ signals), 4.29 (≡C–C$\underline{H_2}$ –O– signals), 4.15 – 3.38 (remaining signals), 2.98 ($\underline{H}$C≡C– signals).
13C NMR (126 MHz, D_2O) δ 100.51, 98.58, 82.42, 79.02, 76.19, 75.30, 74.59, 74.53, 74.21, 73.57, 73.52, 72.99, 72.32, 72.08, 71.85, 71.76, 71.66, 68.35, 61.09 ppm.

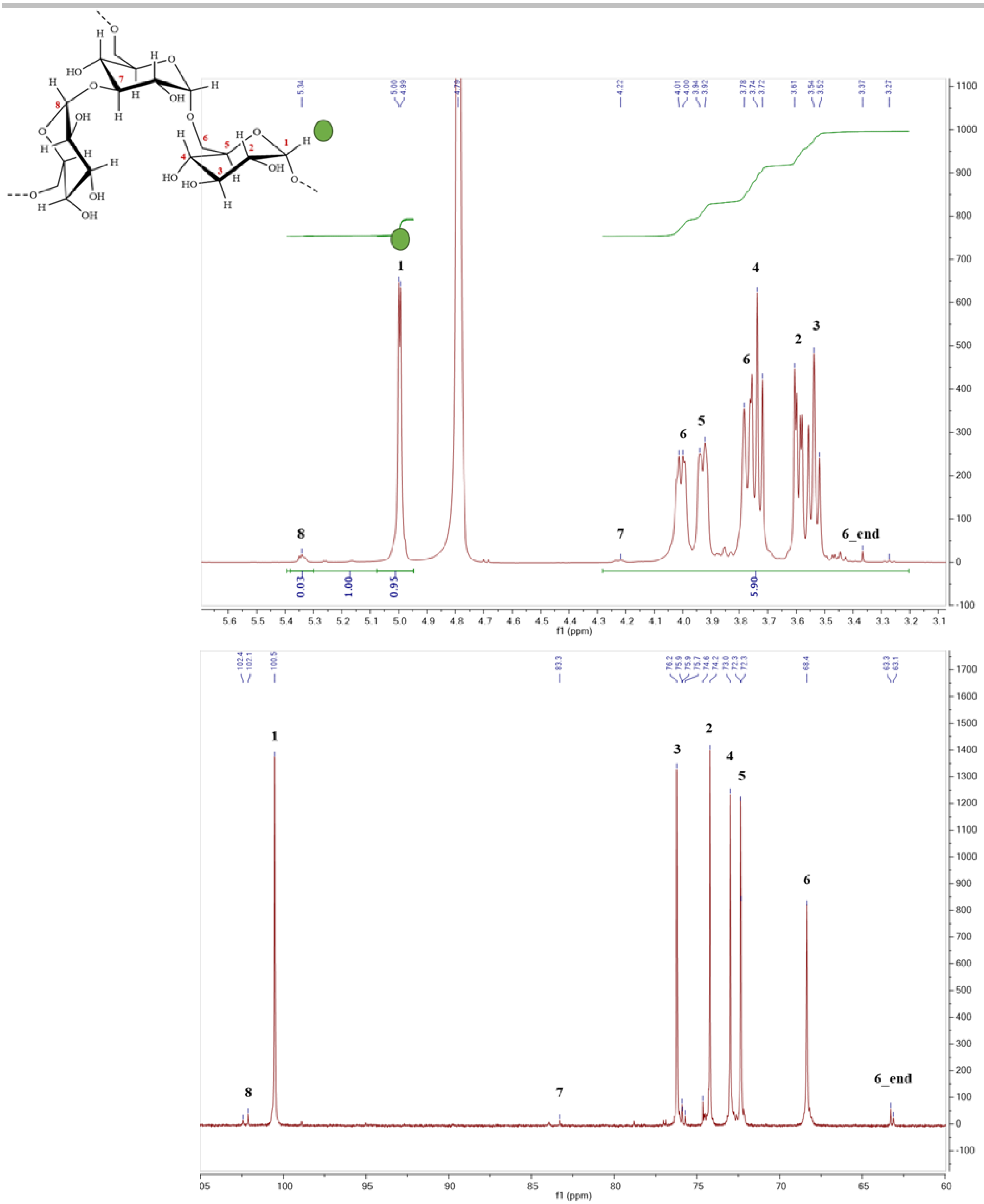

Figure S1 – Recorded NMR spectra (^{1}H up and ^{13}C down) of Dex40 in D_2O with relevant signals assigned.

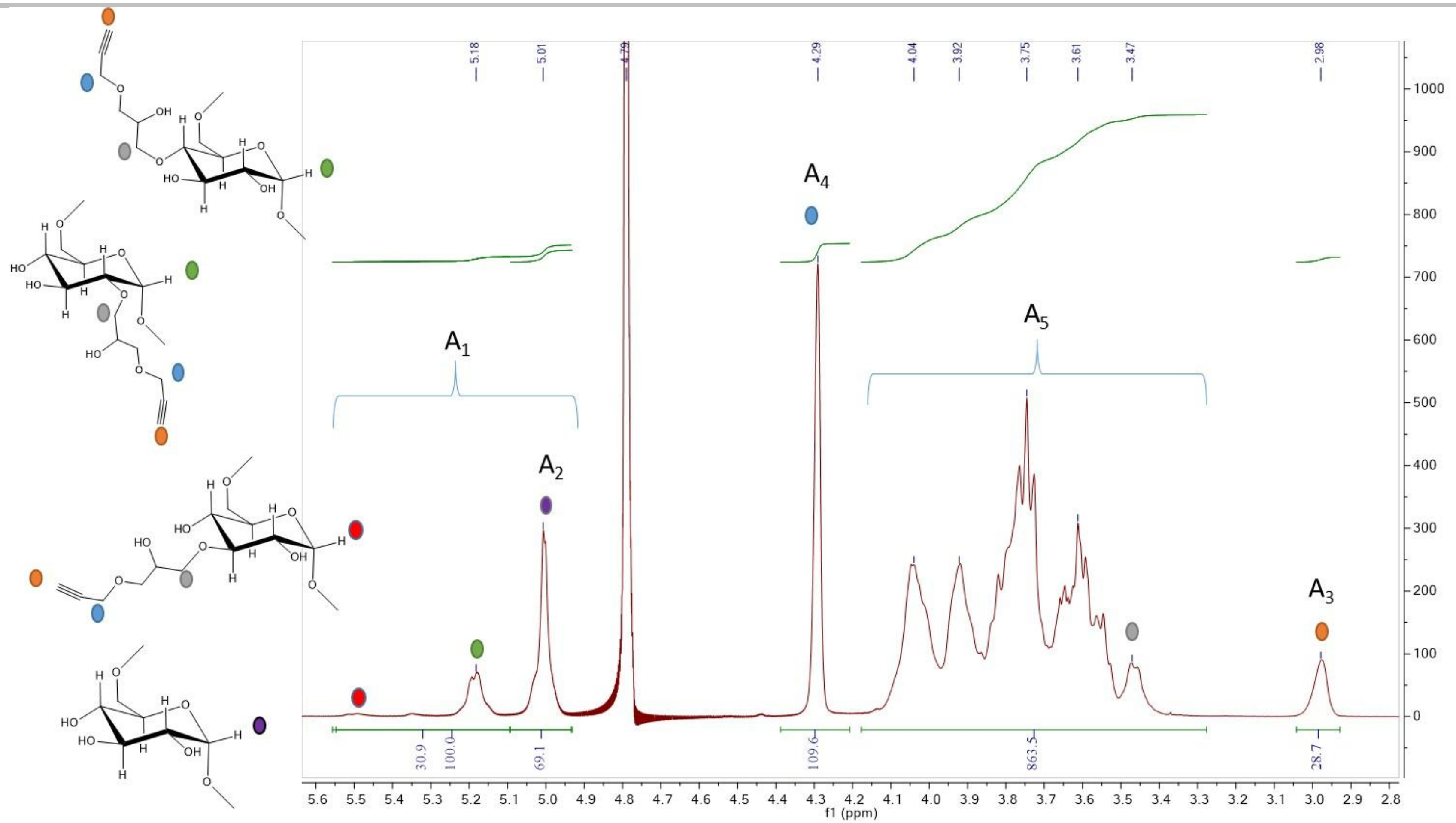

Figure S2 – ^{1}H-NMR spectrum of Dex40-GP with relevant areas used for the characterization, as described in the results section

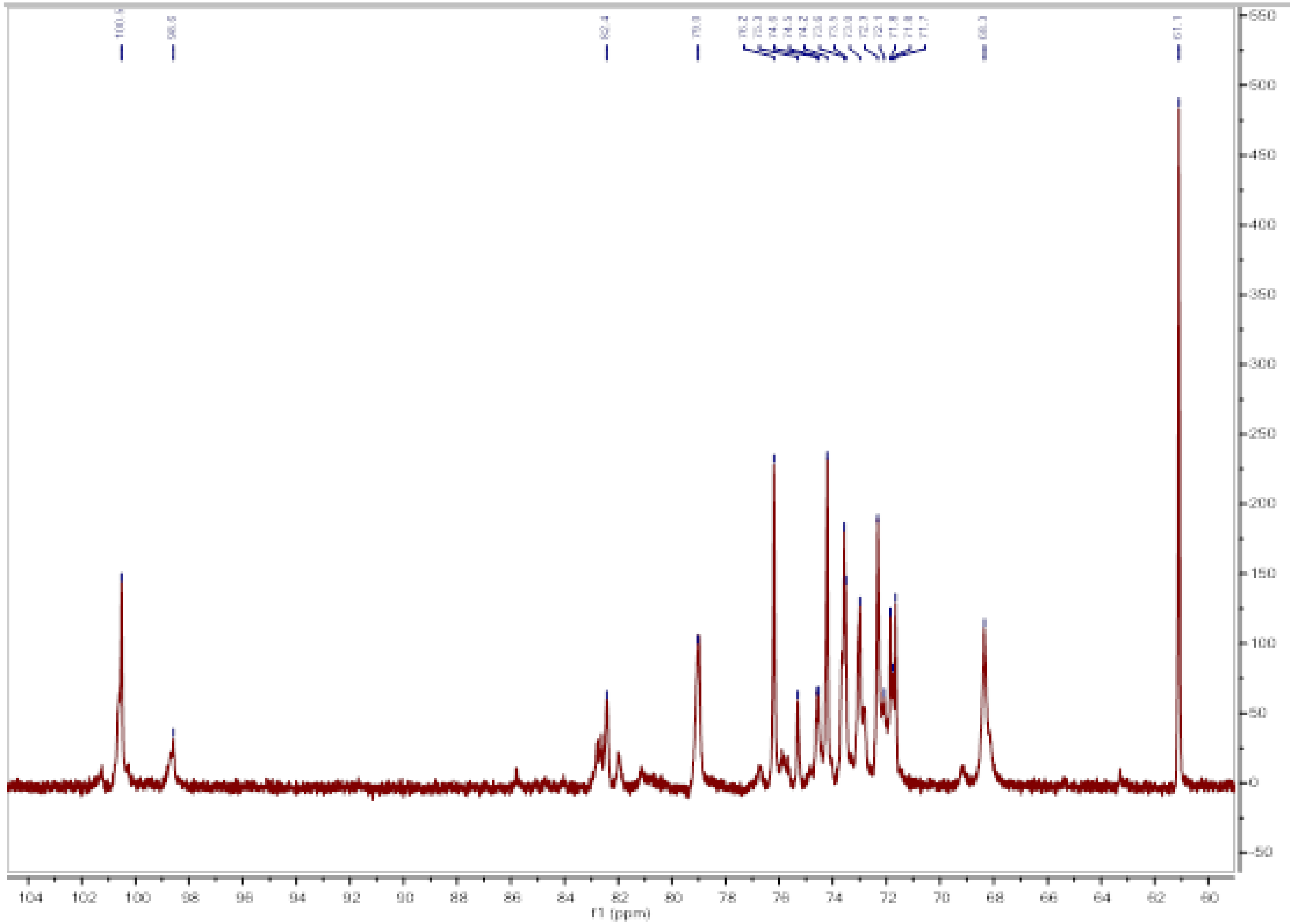

Figure S3 – ^{13}C-NMR spectrum of Dex40-GP. As discussed in the results section, hypothetical assignment of several minor peaks is in agreement with the presence of GP oligomers and alkyne-alkyne couplings.

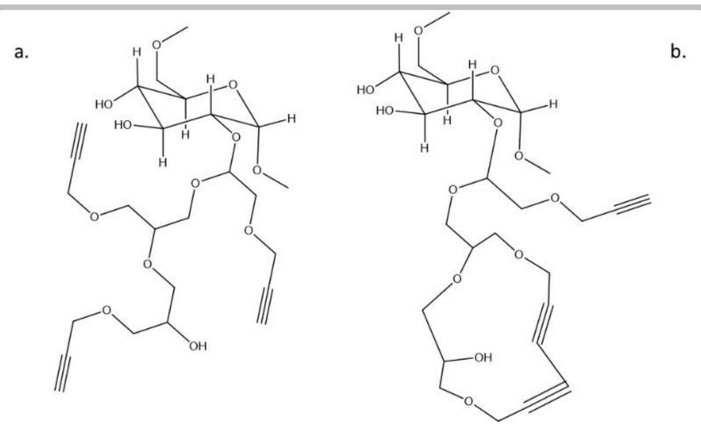

Scheme S1: Oligomerized with n = 3 (a.) and alkyne-alkyne coupled GP functions (b.) on Dex40

2.3.3 NMR of *Peptide 1'$_{A,B}$GK(εN$_3$)*

^{1}H and ^{13}C spectra of peptide **Peptide 1'$_{A,B}$GK(εN3)**, whose structure is shown in Figure S4, were acquired to allow the spectra interpretation of Dex40-Peptide **1'**.

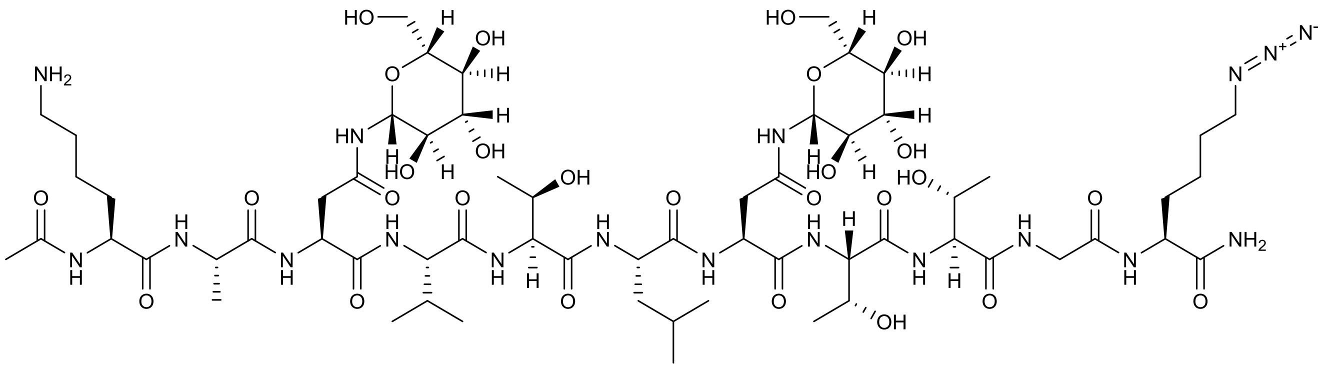

Figure S4 – Structure of Peptide 1'$_{A,B}$GK(εN$_3$): Ac – Lys – Ala – Asn (Glc) – Val – Thr – Leu – Asn (Glc) – Thr – Thr – Gly – Lys(N$_3$) – NH$_2$

The peptide has 80 non-exchangeable protons, but the sum of the integrated signals in ^{1}H spectrum is 78 because the H^{α} signal from the two Asn(Glc) moieties falls in the solvent range (≈ 4.70 ppm) (Figure S5).

^{1}H NMR (500 MHz, D$_2$O) δ 4.97 (d, *J* = 9.2 Hz, 2H, H^{1} Glc), 4.49 – 4.14 (superimposed signals, 11H, H^{α} area + H^{β} Thr), 4.00 (s, 2H, H^{α} Gly), 3.87 (superimposed dd, 2H, H^{6} Glc), 3.74 (dd, *J* = 12.4, 5.1 Hz, 2H, H$^{6'}$ Glc), 3.56 (t, *J* = 9.1 Hz, 2H, H^{3} Glc), 3.50 (m, 2H, H^{5} Glc), 3.44 (t, *J* = 9.4 Hz, 2H, H^{4} Glc), 3.40 (t, *J* = 9.2 Hz, 2H, H^{2} Glc), 3.35 (t, *J* = 6.8 Hz, 2H, H^{ε} Lys(N$_3$)), 3.05 – 2.80 (superimposed signals, 6H, t H^{ε} Lys + dd H^{β} Asn), 2.14 (h, *J* = 6.8 Hz, 1H, H^{β} Val), 2.05 (s, 3H, -CH$_3$ Ac), 1.94 – 1.57 (m, 11H, H^{β1,2} H^{δ} Lys + H^{β1,2} H^{δ} Lys(N$_3$) + H^{β1,2} H^{γ} Leu), 1.54 – 1.41 (m, 4H, H^{γ} Lys + H^{γ} Lys(N$_3$)), 1.40 (d, *J* = 7.2 Hz, 3H, H^{β} Ala), 1.27-1.22 (superimposed d, *J* ≈ 6.4 Hz, 9H, H^{γ2} Thr), 0.97-0.93 (superimposed d, 9H, H^{δ2} Leu + H^{γ} Val), 0.89 (d, *J* = 5.6 Hz, 3H, H^{δ1} Leu).

Peptide **1'N$_3$** possesses 62 C, each one originating a distinct signal in ^{13}C-NMR spectrum, except C^{1} and C^{2} of the two glucoses (superimposed signals) (Figure S6).
^{13}C NMR (126 MHz, d$_2$o) δ 179.5 – 174.0 (14 C, C^{amides}) , 80.0 (2 C, C^{1} glucoses), 78.3 – 69.9 (8 C, C^{2,3,4,5} glucoses) , 67.6 (3 C, C^{β} Thr), 61.3 – 59.9 (6 C, C^{6} glucoses + C^{α} Thr + C^{α} Val), 56.3 – 52.4 (7 C, C^{α} area + C^{ε} Lys(N$_3$)), 45.3 – 39.1 (5 C, C^{α} Gly + C^{ε} Lys + C^{β} Lys + C^{β} Asn), 33.3 – 19.4 (17 C, remaining signals) ppm.

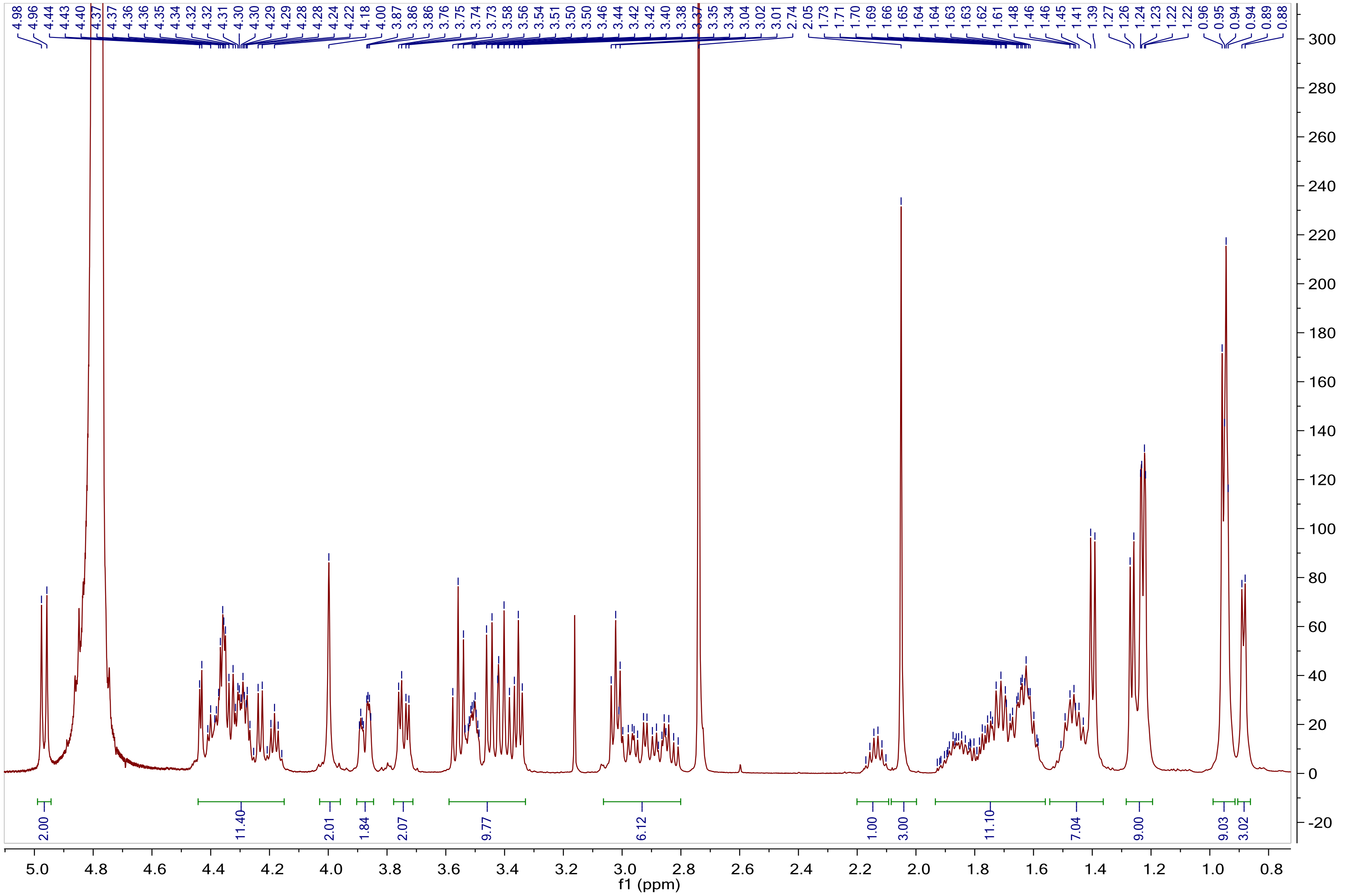

Figure S51 – ^{1}H-NMR spectrum of Peptide 1'$_{A,B}$GK(εN_3)

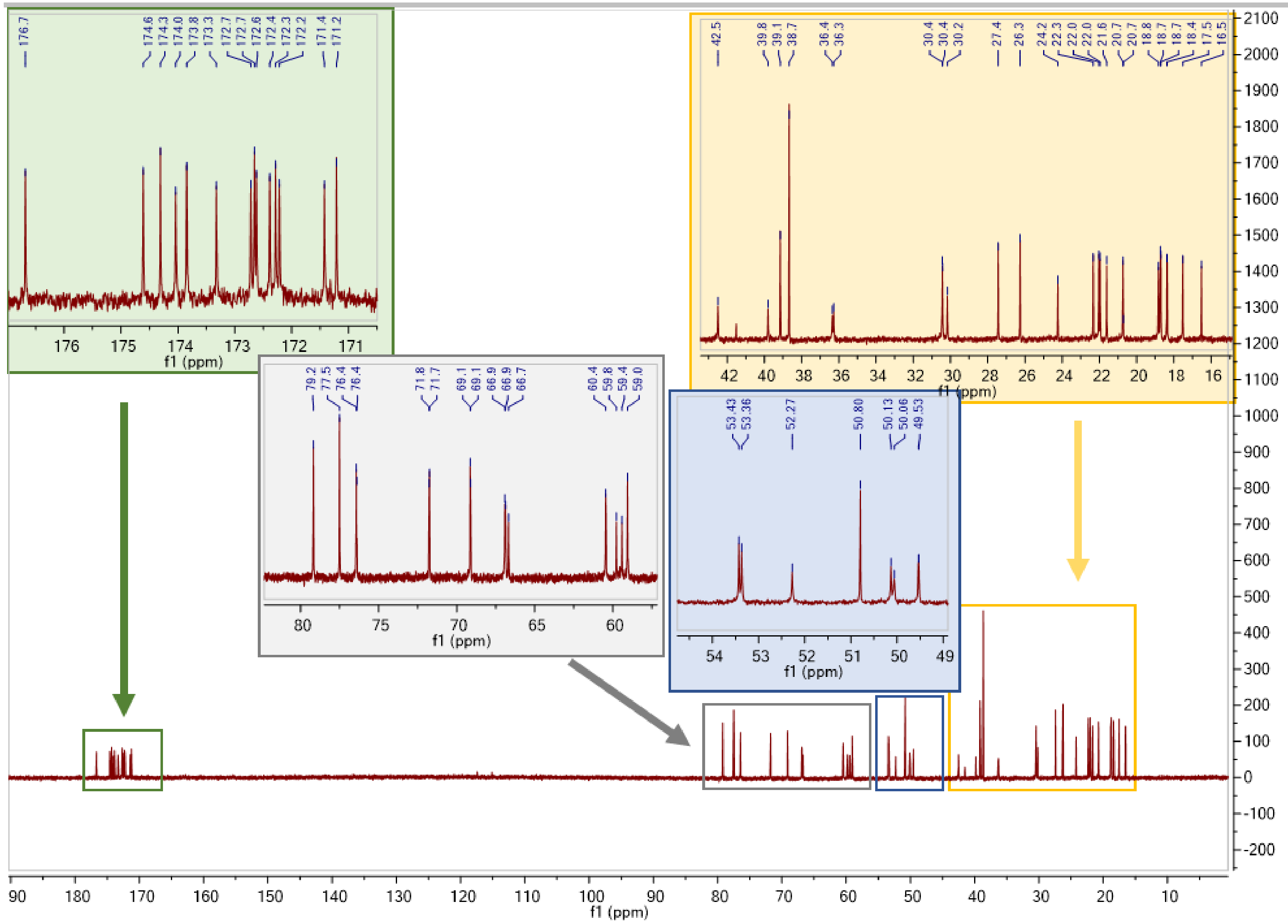

Figure S6 – ^{13}C-NMR spectrum of Peptide 1'$_{A,B}$GK(εN$_3$).

2.3.4 NMR of Dex40-Peptide 1’

Peaks assignment of ^{1}H-NMR spectrum of Dex40-Peptide **1’** was based on previous spectra (Figure S7).
^{1}H NMR (500 MHz, Deuterium Oxide) δ 8.10 (s, 1H, H triazole), 5.31 – 4.95 (m, H^{1} area), 4.54 - 4.19 (m), 4.10 – 3.40 (m), 3.12 – 2.84 (m), 2.20 – 2.16 (m, 1H, H^{β} Val), 2.09 (s, 3H, -CH_3 Ac), 2.04 – 1.35 (m, 18H, $H^{\beta1,2}$ H^{δ} H^{γ} Lys + $H^{\beta1,2}$ H^{δ} H^{γ} Lys(N_3) + $H^{\beta1,2}$ H^{γ} Leu + H^{β} Ala), 1.33-1.23 (m, 9H, $H^{\gamma2}$ Thr), 1.01-0.88 (m, 12H, $H^{\delta1,2}$ Leu + H^{γ} Val).

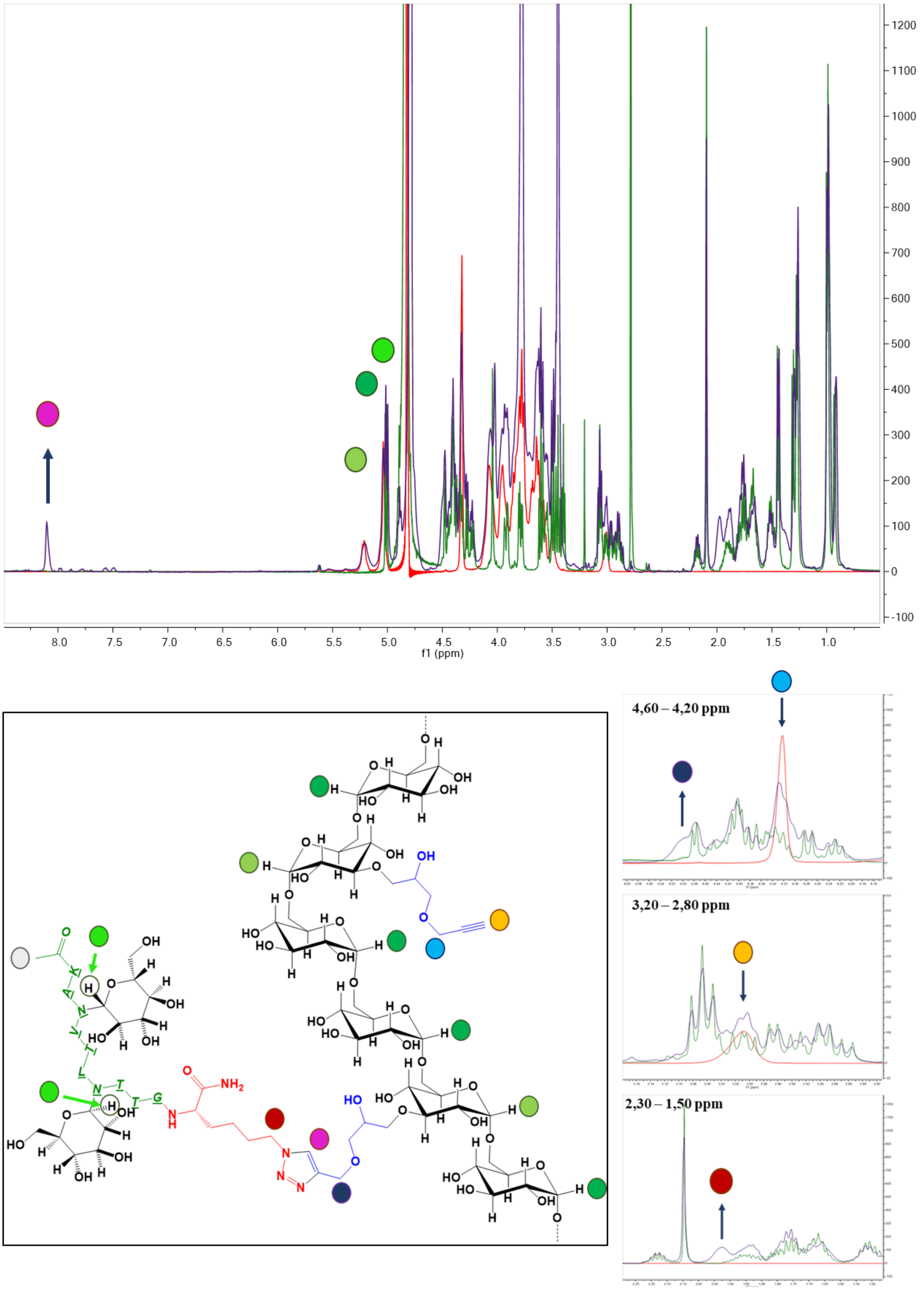

*Figure S7 – Superimposition of ^{1}H-NMR spectra of Dex40-GP (red), Peptide 1'$_{A,B}$GK(εN$_3$) (green) and Dex40-Peptide **1’** (violet). The zoom of relevant ppm ranges shows the diagnostic changes in the spectra after the CuAAC rection (down).*

Relevant integral ratios were used to calculate the DS in peptide and in unreacted alkynyl moieties of Dex40-Peptide 1’. Specifically, ratio between the area of the triazole proton at 8.10 ppm (Figure S8) and different selected signals were used to calculate X ($DS_{Peptide\ 1'}$). In particular:

A) the area of the modified anomeric protons of dextran, A_1, in the 5.15-5.60 ppm range set as 1. The corresponding area of the triazole peak is 0.62 and the $DS_{Peptide\ 1'}$ assumed to correspond to the peak of triazole per GP-modified glucose units in dextran was calculated as follows: 0.62×0.31/1.00 = 0.192 (19.2 %), where 0.31 is the number of the functionalized glucose units in dextran

B) the area of the modified anomeric protons of dextran plus the area due to two protons of the peptide (A_1) in the 5.60-4.95 ppm range set as 1. The corresponding area of the triazole signal is 0.14. The $DS_{Peptide\ 1'}$ was set as X and obtained by the following equation: 0.14/1.00 = X/(1+2X) → X = 0.194 (19.4 %)

C) the area of the 8 Hα + 3 Hβ of peptide residues and methylene protons of GP linkers in the 4.55-4.20 ppm range set as 1. The corresponding area of the triazole peak is 0.06. The $DS_{Peptide\ 1'}$ was set as X and obtained by the following equation: 0.06/1.00 = X/(2×0.31×1.77 +11X) → X = 0.194 (19.4 %)

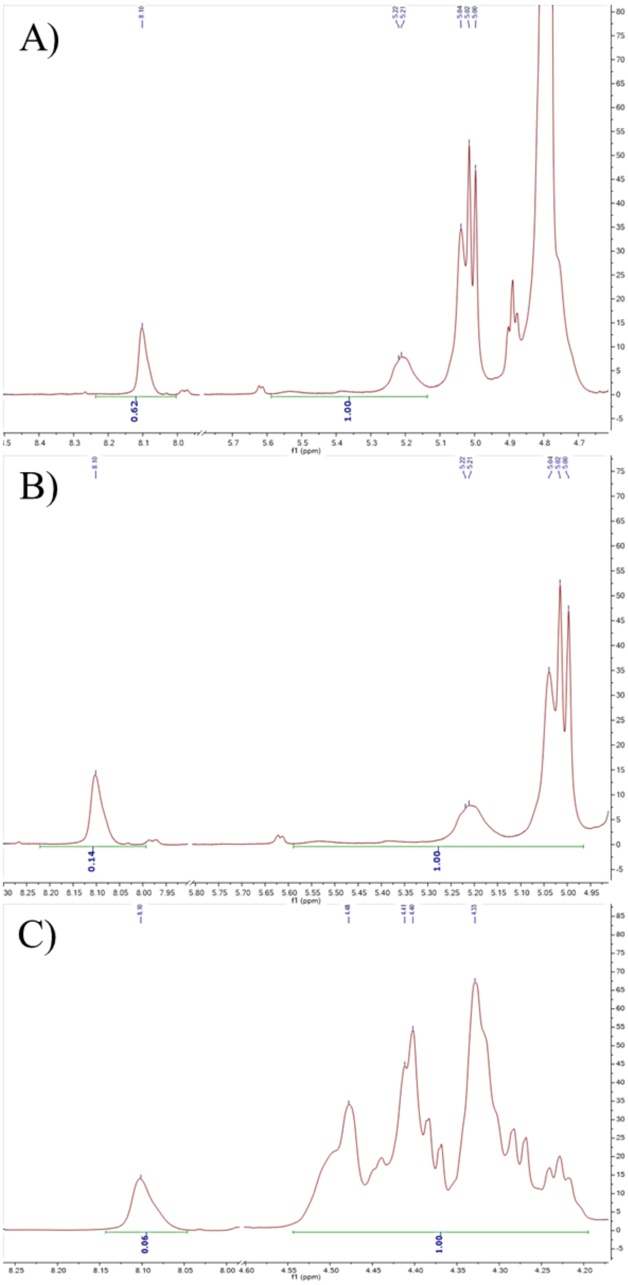

Figure S8 – Zoom of relevant ppm ranges in ^{1}H-NMR spectrum of Dex40-Peptide 1’ used for peptide DS calculation, with corresponding integration values. Triazole proton signal (8.10 ppm) was used as reference in comparison to: A) H1 of modified glucose units (5.60-5.15 ppm); B) H1 of total glucoses, i.e., glucose units of dextran and N-(βGlc) moieties of Peptide $1'_{A,B}GK(\varepsilon N_3)$ (5.60-4.95 ppm); C) the range containing 8 Hα + 3Hβ of peptide residues and methylene protons of GP linkers (4.55-4.20 ppm).

By reasonably varying spectrum parameters such as baseline, phase and integration limits, obtained values for $DS_{peptide\mathbf{6}}$ are always between 19% and 20%, therefore 19.5% was used as practical value for further calculations. Consequently, 9.5% (29-19.5) is accepted as a good estimation of remaining terminal alkynes.
^{13}C NMR (126 MHz, d$_2$o) δ 179.2 – 173.9 (14 C, C^{amides}) , 146.7 (1C, C^{triaz}), 127.7 (1C, C^{triaz}), 100.5 – 61.1 (glucose signals, glycidyl signals, propargyl signals, C^{α} C^{β} Thr, C^{α} Val), 56.3 -19.4 (peptide signals).

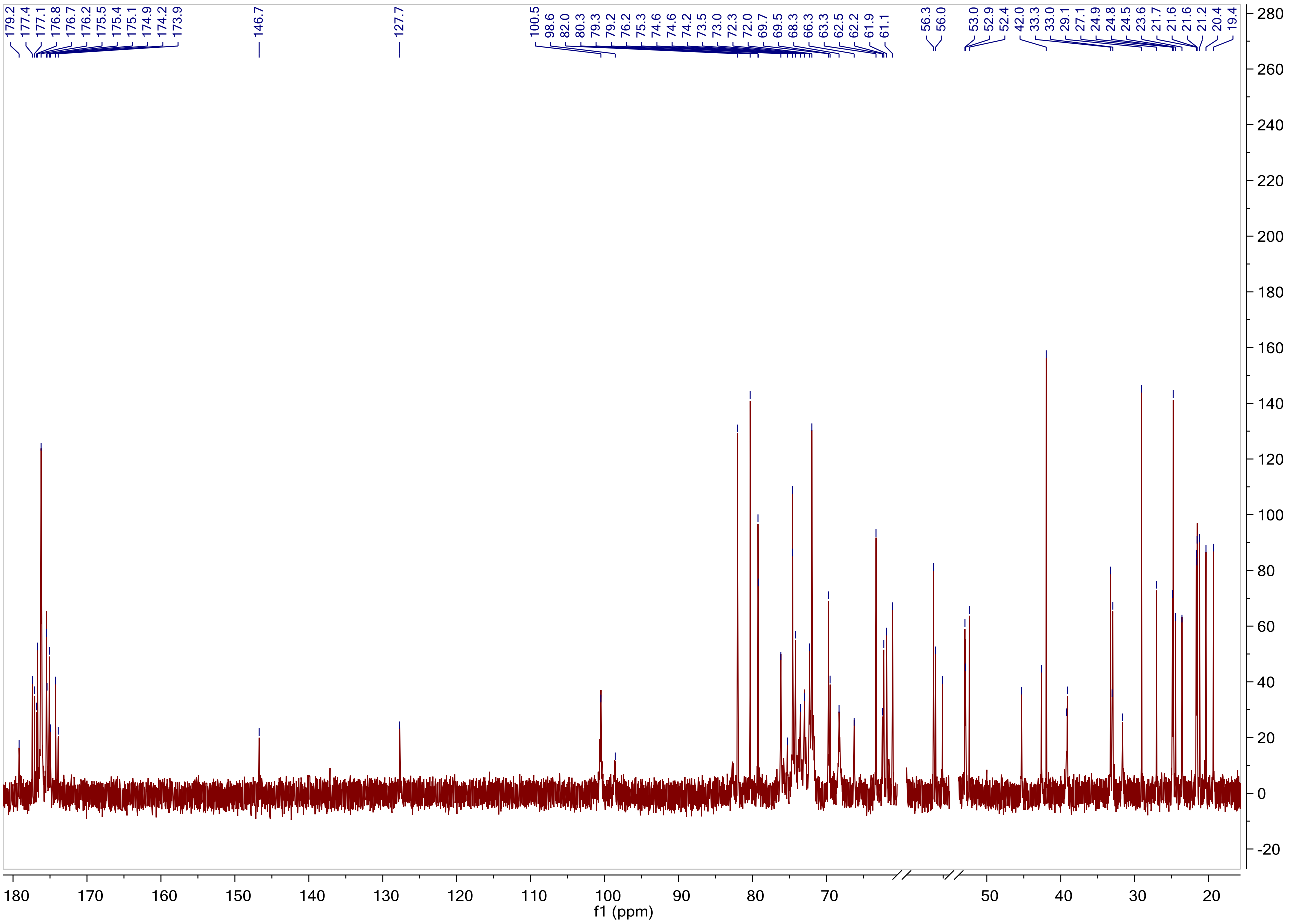

*Figure S9 – ^{13}C-NMR spectrum of Dex40-Pept**1**. The two isolated peaks at 146.7 and 127.7 originate from the triazole ring and are diagnostic for the success of the reaction.*

2.3.5 Molecular weight of Dex40-GP

The molecular weight of Dex40-GP was calculated as follows:

-the average number of repeating units in Dex40 is:

Mn/M_0 = 246.9

where Mn is the average molecular weight of dextran 40 kDa (Dex40); M_0 is the molecular weight of the repeating glucose unit (162 Da).

-based on the Dex40-GP functionalization degree with alkynyl moieties = 31%; 76.5 glucose units are functionalized, while the remaining 170.4 glucose units remain unfunctionalized.

-the average molecular weight of the alkynyl moieties functionalizing Dex40-GP was calculated considering an oligomerization degree n=1.8 (calculated in section 2.3.4) and the results 203.4 Da (113 × 1.8 Da)

Therefore the molecular weight of Dex40 is: (170.4 × 162) + 75.6 × (203.4+162) = 55229 ≈ 55.3 kDa

3. PROTEIN EXPRESSION

3.1 General procedure for protein expression

Protein fragment HMW1ct and the enzyme HMW1C were expressed similarly to a described protocol[3], using E. coli BL21 cells previously engineered with plasmid pET-45b (+) (Novagen), encoding for the fragment HMW1ct and equipped with the gene for carbenicillin resistance, and plasmid pET-24a (+) (Novagen), encoding for the glucosyltransferase enzyme ApHMW1C and equipped with the gene for kanamicin resistance. Cells cultures were prepared using Luria-Bertani (LB) culture soils; the LB medium (SOC) liquid soil was composed by 10 g of tryptone, 5 g of yeast extract and 10 g of NaCl dissolved in 1 L of H_2O Milli-Q, while the LB Agar Plates soil was composed by 2 g of tryptone, 1 g of yeast extract, 2 g of NaCl and 3 g of Agar dissolved in 200 ml of H2O Milli-Q. Stock solutions of antibiotics were prepared in H_2O Milli-Q and stored at -20°C. Working concentration in cell media is 50 µg/mL for kanamycin (only for hyperglucosylated HMW1ct(N-Glc) and 100 µg/mL for carbenicillin. Lysis buffer (pH 7.5) was composed by 5.96 g of HEPES (50 mM), 2.92 g of NaCl (100 mM) and 50 ml of glycerol (10%) dissolved in 0.5 L of H_2O Milli-Q. Cell were coated on Petri dishes (Nunc, ThermoFisher Scientific) with LB Agar Plates soil, containing the antibiotic(s), and incubated overnight at 37°C to allow the growth of the bacterial colonies. The pre-culture phase was performed by picking up one single circular and isolated colony and transferring it in 5 ml of LB medium (SOC) liquid soil containing the antibiotic(s). The solution was incubated overnight at 37°C under shaking. The pre-culture solution was then transferred in 1 L of the same LB medium (SOC) liquid soil containing the antibiotic(s). The solution was incubated under shaking at 37 °C. Cells growth was monitored measuring the optical density at 600 nm (OD_{600}) with an UV instrument (Amersham Biosciences, Little Chalfont, UK). The same LB medium (SOC) liquid soil was used as blank. When the OD value reached 0.6, the induction of the expression was performed adding 1 mL of isopropyl-β-D-1-thiogalactopyranoside (IPTG) (1 mg/mL solution). Cell suspension was incubated overnight at 16°C under shaking. Cells were recovered through centrifugation at 4000 rpm for 30 min at 4°C. The supernatant was removed, and the pellet was suspended in 20 mL of lysis buffer, recentrifuged again and stored at -20°C after supernatant removal.

3.2 General procedure for protein purification

The pellet was suspended in 30 ml of lysis buffer adding 10 µL/g of cells of protease inhibitor (cocktail Set III EDTA-free, Merk). Mechanical lysis of the cell membrane was obtained by using an ultrasonic processor. The lysis solution was then centrifuged for 110 min at 35000 rpm and the supernatant containing the product(s) was recovered. The purification was performed using an Äkta FPLC system. During the first purification step a Hi Trap-His column (HisTrap HP 5 mL, GE Healthcare) was used with the binding buffer A1 for Hi Trap-His (20 mM Tris buffer, 0.5 M NaCl, 30 mM imidazole, pH 7.4) and the elution buffer B1 for Hi Trap-His (20 mM Tris buffer, 0.5 M NaCl, 0.5 M imidazole, pH 7.4).

The conditioning of the column was performed using buffer A1 for 10 minutes. The supernatant containing the products was then injected and eluted with a gradient from 0% to 100% of buffer B1. The UV detector was set to 280 nm and 215 nm. All the fraction obtained were analyzed through Sodium Dodecyl Sulphate - PolyAcrylamide Gel Electrophoresis technique (SDS-PAGE).

The separation of HMW1ct(N-Glc) from ApHMW1C was obtained in the second purification step through the ion exchange technique. A Hi Trap Q-FF column was used. A buffer exchange in order to substitute buffer B1 with binding buffer A2 (20 mM Tris buffer, 20 mM NaCl, pH 8) for Hi Trap Q-FF was performed using Amicon Ultra Centrifugal Filters (MWCO = 10 kDa). The Hi Trap Q-FF column was then conditioned with buffer A2 for 10 minutes. The sample was injected and eluted using a gradient from 0% to 100% of elution buffer B2 (20 mM Tris buffer, 1 M NaCl, pH 8) for Hi Trap Q-FF. The UV detector was set to 280 nm and 215 nm. All the fraction obtained were analyzed through Sodium Dodecyl Sulphate - PolyAcrylamide Gel Electrophoresis technique (SDS-PAGE).

Both HMW1ct and HMW1ct(Glc) were stocked in PBS buffer (8 g of NaCl, 0.2 g of KCl, 1.44 g of Na_2HPO_4 e 0.24 g of KH_2PO_4 dissolved in 1 L of H2O Milli-Q) at -20°C. Their concentration was calculated using the Lambert-Beer law after an absorption measure performed using an UV spectrometer set to a range from 320 and 240 nm.

3.3 General procedure for SDS-PAGE

The SDS-PAGE gel was prepared by depositing between two glasses the running gel 16% solution, composed by 1.6 ml H2O Milli-Q, 4.27 ml 30% acrylamide, 2 ml 1.5M tris buffer pH 8.8, 80 µl 10% SDS, 80 µl 10% ammonium persulfate (APS), 10 µl tetramethylethylenediamine (TEMED). After the polymerization, the stacking gel 4% solution (1.8 ml H_2O Milli-Q, 0.4 ml 30% acrylamide, 0.750 ml 0.5M tris buffer pH 6.8, 30 µl 10% SDS, 30 µl 10% APS, 6 µl TEMED) was deposited above the previous one inserting the comb for the formation of the wells. After the polymerization, the gel was positioned inside the SDS-PAGE apparatus and the tank buffer 1x (100 mL of Tris buffer/Glycine/SDS (10x) in 1 L) was added. 10 µL of each sample were combined with 5 µL of loading buffer 5x (200mM of Tris-Cl (pH 6,8), 400mM of DTT, 8% of SDS, 0,4% of bromophenol blue and 40% of glycerol), treated to 100°C for a few minutes and centrifuged. Each sample was then loaded in the dedicate well. The commercial marker PageRuler Plus Prestained Protein Ladder, 10 to 250 kDa, was used as reference. The electrophoresis was performed for 90 min at 140 mV and subsequently the gel subjected to stain using a Comassie solution (25 ml H_2O Milli-Q, 20 ml MeOH, 5 ml AcOH, 0.05 g Comassie blue dye) for 30 min. In order to remove the excess dye, the stained gel was treated overnight with a destaining solution (700 ml H_2O Milli-Q, 200 ml MeOH, 100 ml AcOH) under gentle shaking.

4 ELISA TEST

ELISA plates, coating conditions, reagent dilutions, buffers, and incubation times were previously tested. Each sample serum was tested in triplicates (three rows/plate) and blanks were performed using FBS buffer instead of sample sera using identical conditions. The absorbance value for each serum was calculated as (mean Abs of serum triplicate) − (mean Abs of blank triplicate). One positive and one negative serum, as references, were included in each plate for further normalization. Each experiment was performed at least twice in different days. Within-assays and between-assays coefficients of variations were below 10%.

4.1 Sample collection

Multiple Sclerosis (MS) patients' sera samples were collected in the Multiple Sclerosis Clinical Care and Research Centre, Department of Neurosciences, Reproductive Sciences and Odontostomatology, Federico II University (Naples, Italy). Sera samples were obtained for diagnostic purposes, from patients and healthy blood donors who had given their informed consent, and stored at −20 °C until use. The present study was conducted in accordance with the Declaration of Helsinki. All experimental protocols performed were approved by the Ethics Committee 2006 and 2017 (protocol n. 120/06 and 160/17, respectively). The MS group consisted of relapsing-remitting MS (RR-MS) patients after a diagnostic lumbar puncture, cerebrospinal fluid analysis, and MRI examination and fulfilled established international diagnostic criteria. Blood samplings in the patients' group were performed during the routine follow-up study, while the healthy control samples were carried out during routine health checks or blood donations.

4.2 Indirect competitive ELISA

Coating antigen was dissolved in H_2O (1 mg/mL) and then diluted 1:100 in carbonate buffer 0.05 M at pH 9.6 (peptides and dextran conjugates) or in PBS pH 7.2 (HMW1 and HMW1ct(Glc). 100 µL of coating antigen solution were added to each well (1 µg antigen/well) of a 96-Well activated Polystyrene ELISA plate (NUNC Maxisorp, ThermoFisher), and incubate overnight at 4°C. The plate was then emptied, washed three times with 0,9% w/w saline solution containing 0.05% v/v Tween 20, then emptied again. 100 µL of Fetal Bovine Serum solution (FBS 10% in saline Tween) /well were added and the plate was left to incubate 2 h at room temperature. Then plate was emptied and a mixture of sera and competing antigen was added in each well. Serum concentration was constant (dilution 1:300) while competing antigen concentration was between 10^{-14} and 10^{-5}.
The plate was incubated 1h at room temperature, then emptied, washed three times, and emptied again.
100 µL of secondary antibody solution (alkaline phosphatase conjugated anti human IgM or IgG Fab2-specific affinity purified antibodies) /well were added and incubated 3 h at room temperature. Each plate was emptied, washed three times then emptied.
100 µL/well of substrate solution (*p*-nitrophenylphosphate 0.1% w/v in carbonate buffer and $MgCl_2$ 10 mM) were added and absorbance was measured at regular intervals with a plate reader (Tecan-Sunrise spectrophotometer working at 405 nm). After 30-60 min, the reaction was blocked by adding 50 µL NaOH 1M/well and the final absorbance value was measured.
Peptides concentration-absorbance relationship was represented graphically as signal inhibition percentage, and half-maximal response concentration values (IC_{50}) were calculated with GraphPad Prism.

4.3 Indirect Solid-phase Elisa (SP-ELISA)

To find the best conditions in order to perform indirect SP-ELISA screening of a larger batch of sera, preliminary tests were carried out for peptides **1'**$_{\mathbf{AB}}$ and a construct of two units of peptide **1'**$_{\mathbf{AB}}$ (**7**), and Dex40-Peptide 1'.
The following parameters were evaluated:

- Presence or absence of the coated antigen
- Coating buffer (Na_2CO_3 0.05 M buffer pH 9.6 or PBS buffer pH 7.2)
- Blocking buffer (10% fetal bovine serum (FBS) or 5 % bovine serum albumin (BSA))
- IgG and IgM detection
- 5 different representative sera + no serum (blank)

ELISA plates, sera dilutions and incubation times were not tested, and used according to previously optimized procedures employing glucosylated antigens for the detection of antibodies in MS sera[83,97].
Briefly, 96-Well activated Polystyrene ELISA plates (NUNC Maxisorp, ThermoFisher) were coated with 1 µg/100 µL/well of antigen in coating buffer (or pure coating buffer without antigen) and incubated at 4 °C overnight. After 3 washes with 0,9% w/w saline solution containing 0.05% v/v Tween 20, non-specific binding sites were blocked with blocking buffer at r.t. for 60 minutes. Sera diluted 1:100 in blocking buffer (100 µL/well) were applied at 4 °C for 16 h. After 3 washes, 100 µL/well of secondary antibody solution (alkaline phosphatase conjugated anti human IgM or IgG Fab2-specific affinity purified antibodies diluted in blocking buffer) were added. After 3 h incubation at room temperature, plates were washed 3 times and then 100 µL/well of substrate solution (*p*-nitrophenylphosphate 0.1% w/v in carbonate buffer and $MgCl_2$ 10 mM) were added. After 15 minutes (IgG plates) or 40 minutes (IgM plates), the reaction was blocked with 50 µL of 1 M NaOH and the absorbance read in a plate reader (SUNRISE, TECAN, Austria) at 405 nm.
Median absorbance values at 405 nm are reported and differentiated according to the different parameters.

5 IMMUNOAFFINITY COLUMN

Preliminary attempts of antibody purification were performed similarly to a previously described method[97] using CNBr-Sepharose resin (Sigma). The resin (100 mg) was washed twice with 1 mL HCl 1 mM and centrifuged at 4000 rpm for 3 minutes. The washing step was repeated three times with H_2O Milli-Q and once with coupling buffer ($NaHCO_3$ 0.1 M, NaCl 0.5 M, pH = 8.3). Resin was then

transferred in a fritted column and washed one last time with coupling buffer (gravity flow). Dex40-Pept**6** (1 mg) was dissolved in 1 mL of coupling buffer and the solution was applied to the resin overnight at room temperature and vigorous shaking. Then the resin was washed with coupling buffer twice, and 1 mL glycine solution (0.2 M, pH = 8.0) was applied to the resin for 2 h at room temperature. The resin was then washed twice with 1 mL coupling buffer, twice with 1 mL acetate buffer (sodium acetate 0.1 M, NaCl 0.5 M, pH = 4.3) and equilibrated with Dulbecco's phosphate buffer saline (D-PBS) at pH = 7.2. Serum MS5 (1 mL) was diluted 1:10 in D-PBS, passed through a 0.22 µm filter and applied to the sepharose column. 10 mL eluted fraction by gravity flow was recirculated one more time through the column and 50 µL from the eluted fraction were taken for ELISA test (FT1 fraction in the results section). The eluted fraction was then applied a third time and left to incubate for 1 h. Final flow through fraction was collected (FT2 fraction in the results section) and then column was washed thoroughly with D-PBS (10 mL) and coupling buffer (10 mL). Adsorbed antibodies were eluted using 10 mL of glycine 0.2 M at pH=2.6. Eluted fraction was immediately neutralized drop-by-drop by adding 10% of $NaHCO_3$ 0.5 M, constantly monitoring the pH with pH paper. The final volume of neutralized eluted fraction is ≈ 15 mL, which is then concentrated by centrifuging with Amicon ultracentrifugal filter units (Merck, MWCO = 50 kDa) and recovered in D-PBS pH = 7.2 (≈1 mL final volume, A_{280} = 0.909). 50 µL of eluted fraction were diluted up to 1 mL in FBS and their activity was checked by SP-ELISA (Elution fraction in the results section). After use, the column was thoroughly washed with additional glycine 0.2 M at pH=2.6 (5 mL), D-PBS buffer (10 mL), coupling buffer (10 mL) and finally with EtOH 20% solution in water, then stored at 4°C to be reused.

References

[1] a) F. Nuti, E. Peroni, F. Real-Fernández, M. A. Bonache, A. Le Chevalier-Isaad, M. Chelli, N. Lubin-Germain, J. Uziel, P. Rovero, F. Lolli, et al., *Biopolymers,* **2010**, *94*, 791–799 b) F. Real Fernández, M. Di Pisa, G. Rossi, N. Auberger, O. Lequin, M. Larregola, A. Benchohra, C. Mansuy, G. Chassaing, F. Lolli, et al., *Biopolymers* **2015**, *104*, 560−576.

[2] H. E. Gottlieb, V. Kotlyar, A. Nudelman, *J. Org. Chem.* **1997**, *62*, 7512‑7515.

[3] M. T. C. Walvoort, C. Testa, R. Eilam, R. Aharoni, F. Nuti, G. Rossi, F. Real-Fernandez, R. Lanzillo, V. Brescia Morra, F. Lolli, et al., *Sci Rep* **2016**, *6*, 39430.
